# A single allele of *Hdac2* but not *Hdac1* is sufficient for normal mouse brain development in the absence of its paralog

**DOI:** 10.1242/dev.100487

**Published:** 2014-02

**Authors:** Astrid Hagelkruys, Sabine Lagger, Julia Krahmer, Alexandra Leopoldi, Matthias Artaker, Oliver Pusch, Jürgen Zezula, Simon Weissmann, Yunli Xie, Christian Schöfer, Michaela Schlederer, Gerald Brosch, Patrick Matthias, Jim Selfridge, Hans Lassmann, Jürgen A. Knoblich, Christian Seiser

**Affiliations:** 1 Department of Medical Biochemistry, Max F. Perutz Laboratories, Medical University of Vienna, Vienna 1030, Austria.; 2 Center for Anatomy and Cell Biology, Medical University of Vienna, Vienna 1090, Austria.; 3 Institute of Pharmacology, Medical University of Vienna, Vienna 1090, Austria.; 4 Institute of Molecular Biotechnology of the Austrian Academy of Sciences (IMBA), Vienna 1030, Austria.; 5 Ludwig Boltzmann Institute for Cancer Research (LBICR), Vienna 1090, Austria.; 6 Division of Molecular Biology, Biocenter Innsbruck, Medical University, Innsbruck 6020, Austria.; 7 Friedrich Miescher Institute for Biomedical Research, Novartis Research Foundation, Basel 4058, Switzerland.; 8 Wellcome Trust Centre for Cell Biology, University of Edinburgh, Edinburgh EH9 3QR, UK.; 9 Center for Brain Research, Medical University of Vienna, Vienna 1090, Austria.

**Keywords:** Chromatin, Epigenetics, Histone deacetylase, Mouse

## Abstract

The histone deacetylases HDAC1 and HDAC2 are crucial regulators of chromatin structure and gene expression, thereby controlling important developmental processes. In the mouse brain, HDAC1 and HDAC2 exhibit different developmental stage- and lineage-specific expression patterns. To examine the individual contribution of these deacetylases during brain development, we deleted different combinations of *Hdac1* and *Hdac2* alleles in neural cells. Ablation of *Hdac1* or *Hdac2* by *Nestin-Cre* had no obvious consequences on brain development and architecture owing to compensation by the paralog. By contrast, combined deletion of *Hdac1* and *Hdac2* resulted in impaired chromatin structure, DNA damage, apoptosis and embryonic lethality. To dissect the individual roles of HDAC1 and HDAC2, we expressed single alleles of either *Hdac1* or *Hdac2* in the absence of the respective paralog in neural cells. The DNA-damage phenotype observed in double knockout brains was prevented by expression of a single allele of either *Hdac1* or *Hdac2*. Strikingly, *Hdac1*^−/−^*Hdac2*^+/−^ brains showed normal development and no obvious phenotype, whereas *Hdac1*^+/−^*Hdac2*^−/−^ mice displayed impaired brain development and perinatal lethality. *Hdac1*^+/−^*Hdac2*^−/−^ neural precursor cells showed reduced proliferation and premature differentiation mediated by overexpression of protein kinase C, delta, which is a direct target of HDAC2. Importantly, chemical inhibition or knockdown of protein kinase C delta was sufficient to rescue the phenotype of neural progenitor cells *in vitro*. Our data indicate that HDAC1 and HDAC2 have a common function in maintaining proper chromatin structures and show that HDAC2 has a unique role by controlling the fate of neural progenitors during normal brain development.

## INTRODUCTION

Epigenetic mechanisms including post-translational modifications of histones and methylation of DNA are essential for activation, repression and fine-tuning of gene expression (Jaenisch and Bird, 2003). Histone acetylation, generally associated with transcriptional activation, is reversibly regulated by histone acetyltransferases (HATs) and histone deacetylases (HDACs). HATs induce the local opening of chromatin regions, whereas HDACs mediate chromatin compaction and transcriptional repression. The classical view of HATs as co-activators and HDACs as co-repressors of transcription has been challenged recently, as HDACs colocalized with HATs on actively transcribed genes, but were not detected on silent genes by genome-wide mapping techniques (Wang et al., 2009). Hence, dynamic and reversible histone acetylation seems to be a prerequisite for modulating the expression of active genes.

In mammals, 18 HDACs have been identified and are grouped into four different classes according to homology and function: class I (HDAC1, HDAC2, HDAC3 and HDAC8), class II (HDAC4, HDAC5, HDAC6, HDAC7, HDAC9 and HDAC10), sirtuin class III, and class IV (HDAC11) (Bolden et al., 2006). The Rpd3-like class I members HDAC1 and HDAC2 are highly homologous (Tsai and Seto, 2002) and are able to homo- and heterodimerize (Taplick et al., 2001). The two paralogs are often found in the same multisubunit repressor complexes including SIN3, CoREST, NuRD, NODE and MiDAC (Alland et al., 1997; Heinzel et al., 1997; Laherty et al., 1997; Zhang et al., 1997; Ballas et al., 2001; Liang et al., 2008; Bantscheff et al., 2011). In addition to histones, class I HDACs deacetylate a variety of proteins including transcription factors and other cellular regulators (Glozak et al., 2005; Peserico and Simone, 2011).

Inhibition of HDACs with small-molecule inhibitors is a promising strategy in the treatment of diseases including pathological conditions of the central nervous system (CNS) and has exhibited beneficial effects in several models of brain disorders (reviewed by Langley et al., 2005; Kazantsev and Thompson, 2008). However, the impact of individual HDACs on certain neurological diseases is not yet resolved. During the last decade, mouse genetics has been successfully used to analyze the functions of HDAC1 and HDAC2 during differentiation and development. We have previously shown that germline deletion of *Hdac1* results in embryonic lethality as a result of proliferation defects and impaired development (Lagger et al., 2002). Conventional deletion of *Hdac2* led to perinatal lethality, partial embryonic lethality or partial lethality during the first few months, depending on the knockout strategy (Montgomery et al., 2007; Trivedi et al., 2007; Zimmermann et al., 2007; Guan et al., 2009; Reichert et al., 2012). These results indicate divergent functions of the two paralogs during mouse embryogenesis. By contrast, conditional loss-of-function studies of *Hdac1* or *Hdac2* in different tissues and cell types have demonstrated redundant functions of HDAC1 and HDAC2 in differentiation and tissue homeostasis (Montgomery et al., 2007; Yamaguchi et al., 2010; Chen et al., 2011; Jacob et al., 2011; Ma et al., 2012).

In the CNS of adult mice, HDAC1 and HDAC2 display exceptional cell type-specific expression patterns (MacDonald and Roskams, 2008) compared with other tissues. HDAC1 is preferentially expressed in astrocytes, whereas HDAC2 shows high expression in mature neurons, while both enzymes are co-expressed in neural precursors during embryogenesis. Deletion of either *Hdac1* or *Hdac2* in a subset of neural precursors and mature astrocytes by *Gfap-Cre* did not affect brain development, whereas combined loss led to severely impaired brain architecture and lethality by postnatal day (P) 7 suggesting functional redundancy of these class I deacetylases (Montgomery et al., 2009). To dissect the individual roles of HDAC1 and HDAC2 in neural development, we have conditionally deleted different combinations of *Hdac1* and *Hdac2* alleles in the nervous system using *Nestin*-*Cre* transgenic mice. Our results identify HDAC2 as the essential class I deacetylase for brain development and survival.

## RESULTS

### Overlapping and distinct expression patterns of HDAC1 and HDAC2 in the murine brain

Originating from a gene duplication, the genes encoding the mammalian class I histone deacetylases HDAC1 and HDAC2 show highly conserved exon-intron structures but are located on different chromosomes (Zeng et al., 1998; Khier et al., 1999). HDAC1 and HDAC2 proteins share 86% amino acid identity and associate with the same transcriptional repressor complexes, suggesting a certain functional redundancy (Brunmeir et al., 2009). However, a notable example of specific roles for HDAC1 and HDAC2 is in the brain, where both enzymes display different developmental stage- and lineage-specific expression patterns (MacDonald and Roskams, 2008). During embryogenesis HDAC1 and HDAC2 showed overlapping expression in different brain regions such as the cortex (supplementary material Fig. S1A). Quantitative immunoblot analysis of P0 brain protein extracts detected modestly elevated HDAC1 levels when compared with HDAC2 (supplementary material Fig. S1B).

**Fig. 1:**
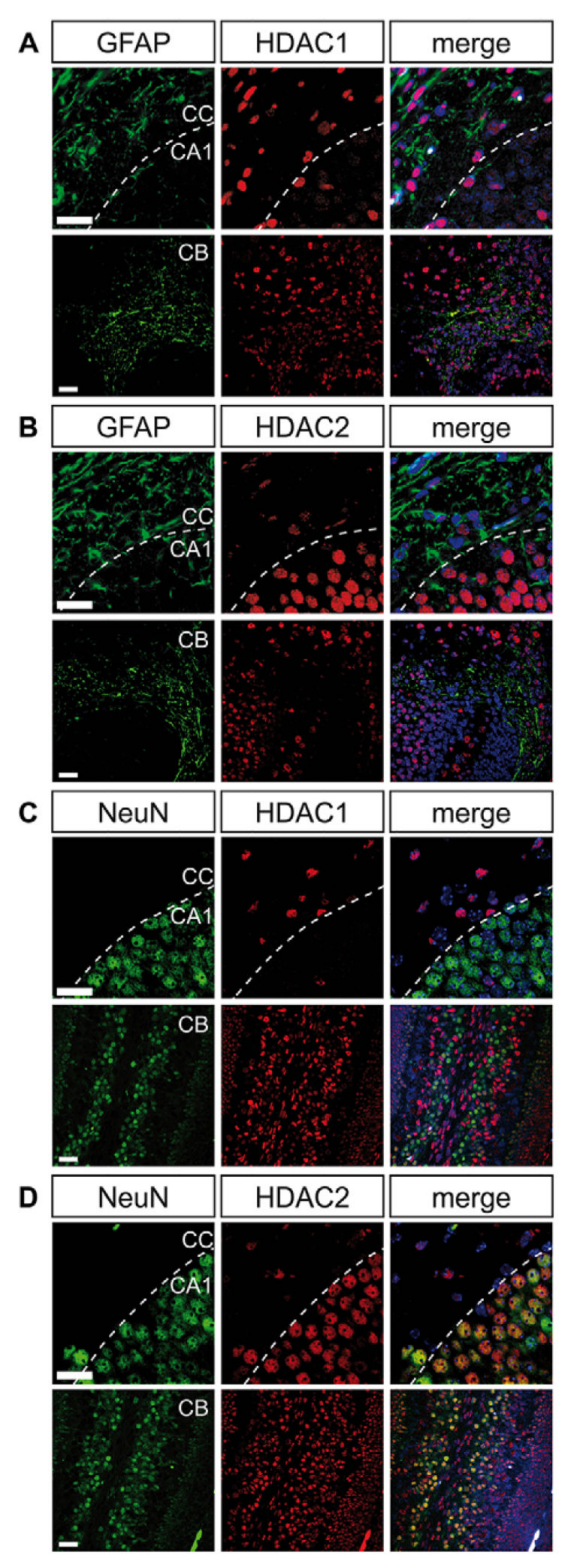
HDAC1 and HDAC2 display divergent expression patterns in the postnatal wild-type brain Fluorescence immunohistochemistry stainings of HDAC1 and HDAC2 in the corpus callosum and the CA1 neuron region of the hippocampus (upper panels) and in the cerebellum (lower panels) on postnatal day 4 (P4). (A,B) Co-staining of astrocyte marker GFAP (green) and HDAC1 (red, A) or HDAC2 (red, B). (C,D) Co-staining of neuronal marker NeuN (green) and HDAC1 (red, C) or HDAC2 (red, D). Nuclei are counterstained with 4′6-diamidino-2-phenylindole (DAPI). The white dashed line indicates the border between the corpus callosum and the CA1 region. Scale bar: 20 μm. CA1, hippocampal CA1 region; CB, cerebellum; CC, corpus callosum.

In the postnatal mouse brain (P4), HDAC1, but not HDAC2, was highly expressed in glial fibrillary acidic protein (GFAP)-positive astrocytes in the corpus callosum (CC) (Fig. 1A,B, upper panels). By contrast, HDAC2, but not HDAC1, was primarily expressed in hippocampal CA1 neurons detected by the neuronal marker neuronal nuclei (NeuN) (Fig. 1C,D, upper panels). The same exclusive HDAC1/HDAC2 expression pattern was observed in other brain regions such as cerebellum (Fig. 1, lower panels), cortex, medulla at P4 and in the adult brain (data not shown). We therefore conclude that from P4 onwards HDAC1 is mainly expressed in astrocytes and HDAC2 is predominantly expressed in neurons, except for rare mature neurons and embryonic progenitor cells. Given that HDAC1 and HDAC2 are designated transcriptional co-regulators, we next asked whether their expression was determined by a negative feedback loop controlled by the paralog enzyme. This mechanism would result in exclusive mRNA expression in either neurons or astrocytes. However, the regulatory crosstalk is more likely to occur on translational or post-translational levels, as neuron-rich and astrocyte-rich brain areas obtained by laser microdissection showed similar mRNA expression levels for both *Hdac1* and *Hdac2* despite differential cell type-specific protein expression (supplementary material Fig. S2).

### Deletion of either *Hdac1* or *Hdac2* leads to re-expression of the respective paralog and does not affect overall brain anatomy

**Fig. 2:**
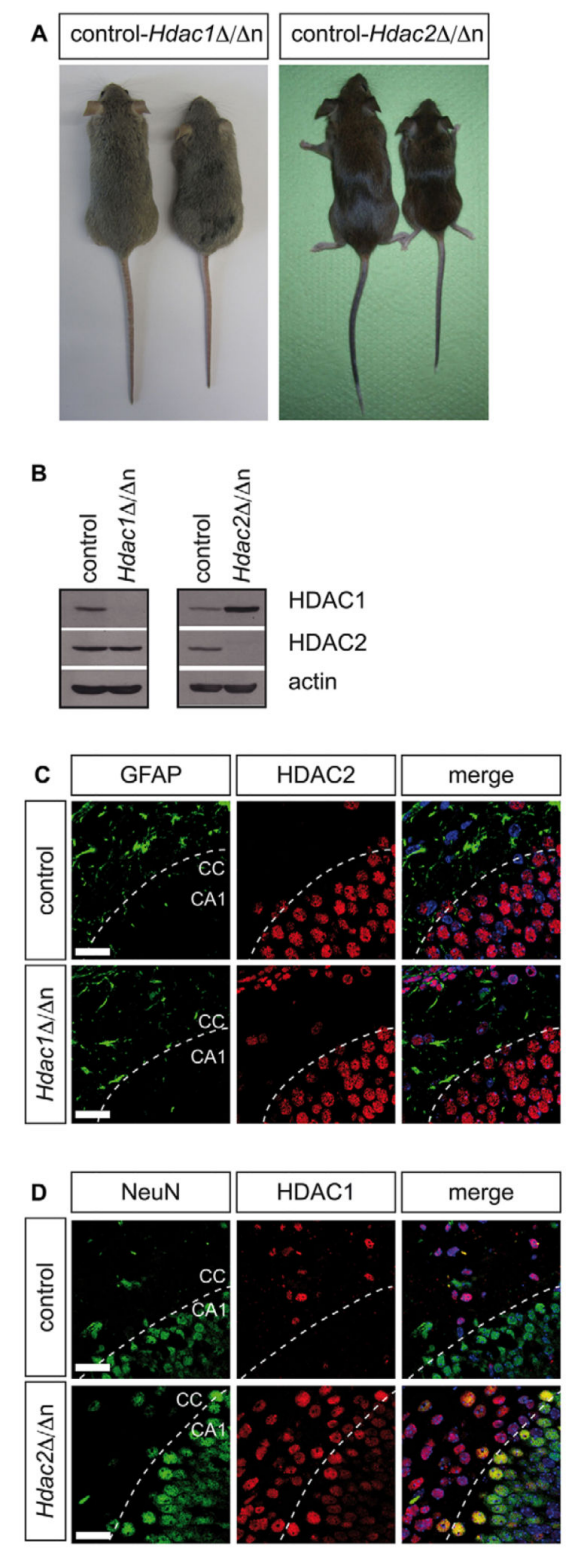
Deletion of either *Hdac1* or *Hdac2* leads to expression of its paralog in the brain (A) Left panel: representative pictures of a wild-type (left) versus an *Hdac1*^Δ/Δn^ (right) adult littermate. Right panel: pictures of a wild-type (left) versus an *Hdac2*^Δ/Δn^ (right) adult littermate. (B) Immunoblot analyses of P0 wild-type littermate controls versus *Hdac1*^Δ/Δn^ (left panel) and *Hdac2*^Δ/Δn^ (right panel) brain extracts. The membrane was probed with antibodies against HDAC1, HDAC2 and β-actin as loading control. (C) Fluorescent immunohistochemistry (IHC) stainings of GFAP (green) and HDAC2 (red) in P4 *Hdac1*^Δ/Δn^ (lower panel) and wild-type littermate control (upper panel) mice in the corpus callosum and the CA1 region of the hippocampus. (D) Fluorescent IHC stainings of NeuN (green) and HDAC1 (red) in P4 *Hdac2*^Δ/Δn^ (lower panel) and wild-type littermate control (upper panel) mice. Nuclei are counterstained with DAPI. The white dashed line indicates the border between the corpus callosum and the CA1 region. Scale bar: 20 μm. CA1, hippocampal CA1 region; CC, corpus callosum.

As the cell type-specific expression pattern suggested distinct and independent functions for HDAC1 and HDAC2, we aimed to study their individual contribution to mouse brain development. To generate mice lacking either HDAC1 or HDAC2 in the nervous system, we crossed mice with floxed *Hdac1* or *Hdac2* alleles (referred to as *Hdac1*^f/f^ or *Hdac2*^f/f^) to transgenic mice expressing Cre recombinase under the control of the rat nestin (*Nes*) promoter and enhancer (Tronche et al., 1999). The *Nestin-Cre* transgene is permanently activated in neural precursors from embryonic day (E) 9.5 to E11 and results in HDAC1 or HDAC2 deficiency in all major cell types of the nervous system (hence referred to as *Hdac1*^Δ/Δn^ and *Hdac2*^Δ/Δn^, respectively). In accordance with a previous study using the *Gfap-Cre* transgene (Montgomery et al., 2009), *Nestin-Cre*-mediated deletion of either *Hdac1* or *Hdac2* caused reduced body size and weight (Fig. 2A; supplementary material Fig. S6A), but resulted in no overall change in brain histoarchitecture or lifespan (data not shown). Absence of HDAC2 led to upregulation of HDAC1 protein, whereas HDAC2 levels showed no obvious changes in *Hdac1*^Δ/Δn^ brains (Fig. 2B). Remarkably, *Hdac1*^Δ/Δn^ mice expressed HDAC2 protein in astrocytes (Fig. 2C) whereas *Hdac2*^Δ/Δn^ mice displayed expression of HDAC1 in neurons (Fig. 2D). These results reveal a mechanistic crosstalk of HDAC1 and HDAC2 in neurons and astrocytes and strongly suggest sufficient compensation to prevent CNS abnormalities.

### Combined deletion of *Hdac1* and *Hdac2* leads to severely impaired brain development and embryonic lethality

**Fig. 3:**
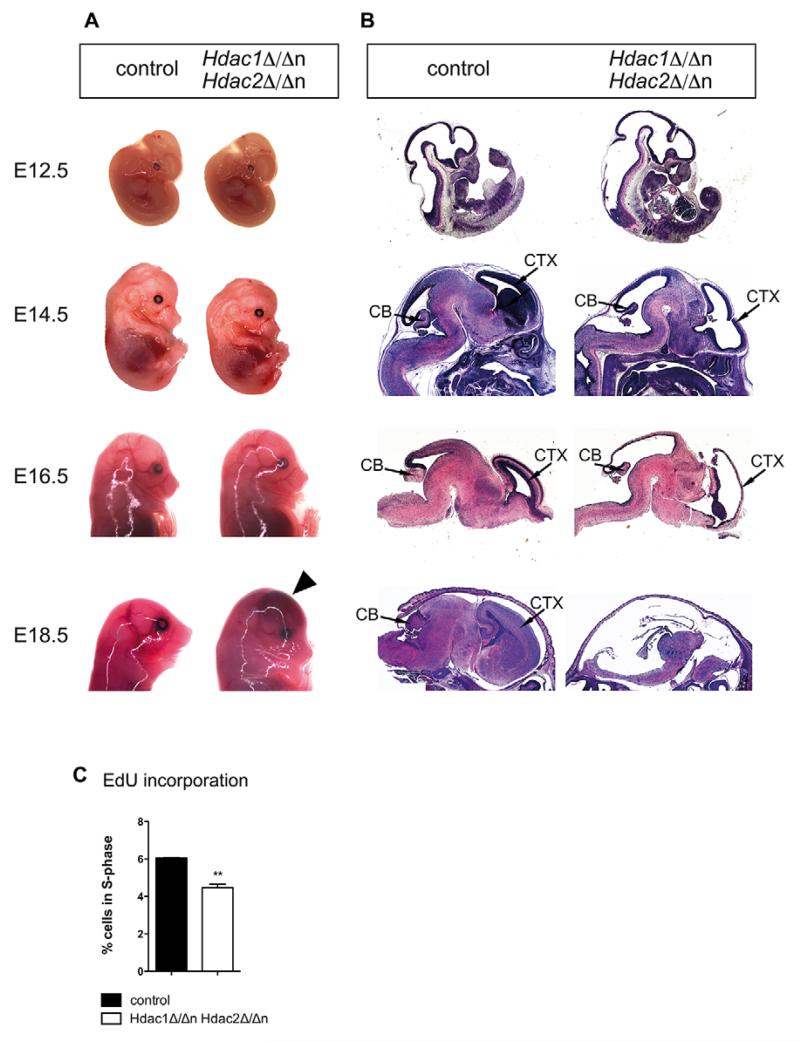
Combined deletion of *Hdac1* and *Hdac2* in the nervous system leads to embryonic lethality (A) Representative pictures of *Hdac1*^Δ/Δn^*Hdac2*^Δ/Δn^ (right) and wild-type littermate controls (left) at consecutive embryonic time points (E12.5, E14.5, E16.5 and E18.5). The black arrowhead indicates a region affected by brain hemorrhage. (B) Hematoxylin and Eosin stainings of *Hdac1*^Δ/Δn^*Hdac2*^Δ/Δn^ (right) and wild-type littermate control representative paraffin sections (left) at indicated embryonic time points (E12.5, E14.5, E16.5 and E18.5). Cortex and cerebellum are indicated. (C) Quantification of S-phase cells monitored by 5-ethynyl-2′-deoxyuridine (EdU) incorporation and subsequent fluorescence-activated cell sorting (FACS) analysis in E14.5 *Hdac1*^Δ/Δn^*Hdac2*^Δ/Δn^ (white) and control littermate (black) brains. Error bars indicate s.d. (*n*=3). **P<0.01. CB, cerebellum; CTX, cortex.

**Fig. 4:**
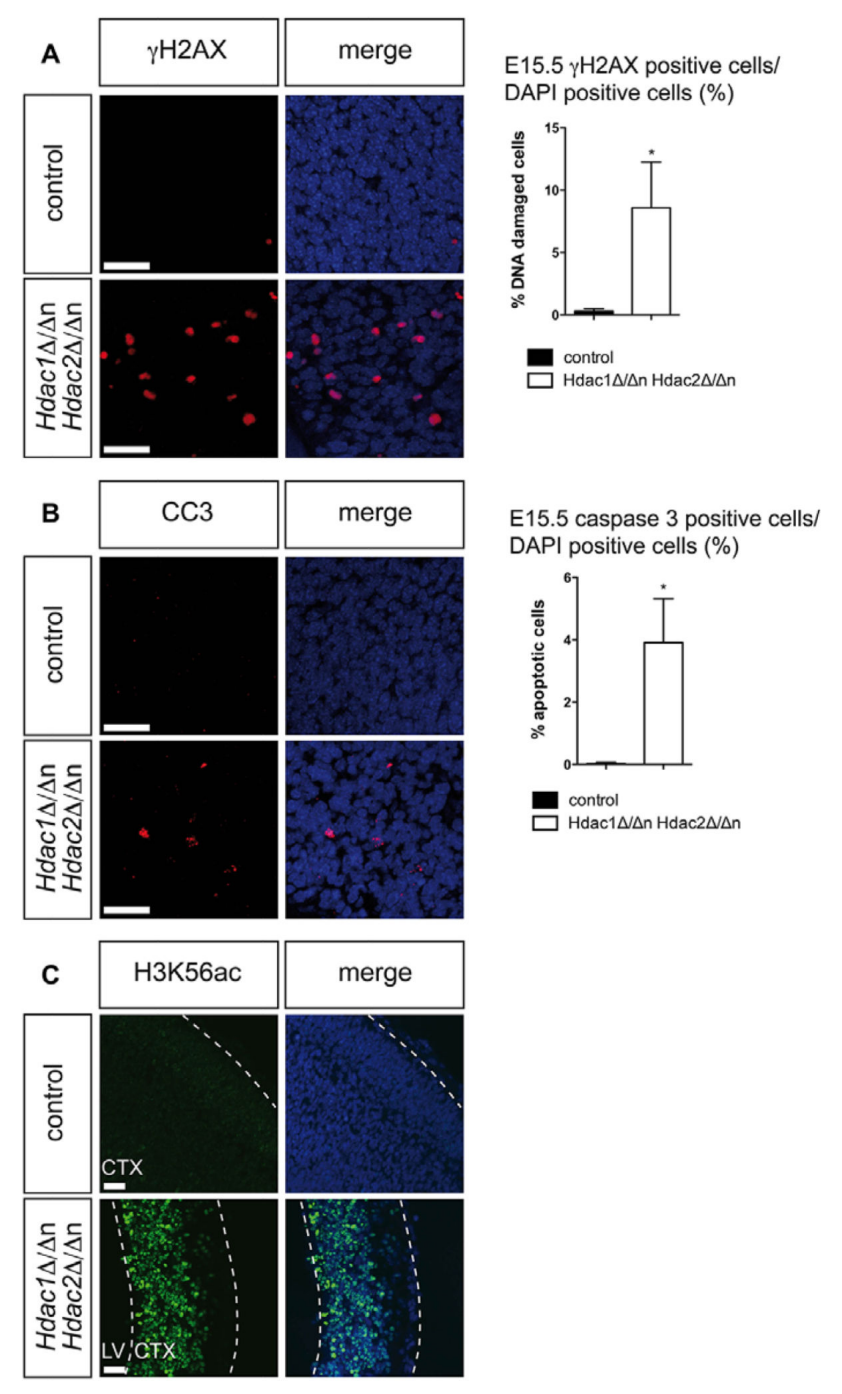
Combinatorial loss of HDAC1 and HDAC2 results in increased DNA damage and apoptosis at E15.5 (A) Fluorescent IHC stainings of γH2AX (red) on *Hdac1*^Δ/Δn^*Hdac2*^Δ/Δn^ brains (lower panel) and wild-type littermate controls (upper panel). (B) Fluorescent IHC stainings of cleaved caspase 3 (red) on *Hdac1*^Δ/Δn^*Hdac2*^Δ/Δn^ brains (lower panel) and wild-type littermate controls (upper panel). (C) Fluorescent IHC stainings of H3K56ac (green) on *Hdac1*^Δ/Δn^*Hdac2*^Δ/Δn^ brains (lower panel) and wild-type littermate controls (upper panel). For quantification, positively stained cells in *Hdac1*^Δ/Δn^*Hdac2*^Δ/Δn^ (white) and the corresponding wild-type control mice (black) were counted as shown in the graphs on the right. Error bars indicate s.d. (*n*=2). *P<0.05. Nuclei are counterstained with DAPI. Scale bars: 20 μm. CC3, cleaved caspase 3; CTX, cortex; LV, lateral ventricle.

Given the compensatory cross-regulation of HDAC1 and HDAC2, we generated mice with simultaneous ablation of both enzymes in the nervous system (*Hdac1*^Δ/Δn^*Hdac2*^Δ/Δn^). The lack of both proteins was confirmed by immunoblot analysis and immunohistochemistry (IHC) (supplementary material Fig. S3B,C). Simultaneous ablation of *Hdac1* and *Hdac2* resulted in severely compromised brain development and death before birth (supplementary material Fig. S3A). When compared with wild-type littermates, reduced cellular proliferation and smaller sizes of cortex and cerebellum became evident at E14.5. Later time points of analysis indicated progressive aggravation of the phenotype, culminating in degeneration and almost entire loss of brain tissue at E18.5 (Fig. 3B). Moreover, *Hdac1*^Δ/Δn^*Hdac2*^Δ/Δn^ brains exhibited severe cerebral hemorrhage, detectable in embryo whole mounts as early as E18.5 (Fig. 3A). At E14.5 we observed reduced proliferation (Fig. 3C) and increased DNA damage, which was not accompanied by activation of apoptosis at this stage (supplementary material Fig. S3D,E). By contrast, at E15.5, *Hdac1*^Δ/Δn^*Hdac2*^Δ/Δn^ brains exhibited both increased DNA damage and apoptosis (Fig. 4A,B; supplementary material Fig. S3F,G). Morphologic analysis of *Hdac1*^Δ/Δn^*Hdac2*^Δ/Δn^ brains at the ultrastructural level demonstrated typical stratification of the cortex at E15.5 (supplementary material Fig. S4), although total cell numbers were found to be decreased in the outer layers. In *Hdac1*^Δ/Δn^*Hdac2*^Δ/Δn^ mice the subventricular layer appeared to be heterogeneous in cell composition and contained nuclei with morphologic characteristics of more outer layers (supplementary material Fig. S4G). We further observed increased signs of cell death in the subventricular and intermediate zones of *Hdac1*^Δ/Δn^*Hdac2*^Δ/Δn^ mice, illustrated by cells containing phagosomes and the presence of cell debris (supplementary material Fig. S4G).

In order to examine chromatin-associated consequences of *Hdac1*/*Hdac2* ablation we measured total cellular deacetylase activity and examined the levels of individual histone acetylation marks. Simultaneous loss of HDAC1 and HDAC2 resulted in strong reduction of total cellular deacetylase activity (supplementary material Fig. S5A) and concomitant increase in specific histone acetylation marks (supplementary material Fig. S5C,D). In particular, acetylation levels for H3K4, H3K9, H3K14, H3K27, H3K56, H4K8 and H4K16 were significantly increased upon combined deletion of *Hdac1* and *Hdac2* (Fig. 4C; supplementary material Fig. S5C,D).

Gene expression profiling revealed 1546 deregulated genes in *Hdac1*^Δ/Δn^*Hdac2*^Δ/Δn^ brains (at least twofold deregulation, *P*<0.05) with a majority of upregulated genes (supplementary material Fig. S5B; supplementary material Table S1). Functional gene ontology (GO) analysis identified the highest percentage of upregulated genes as immune-response genes (supplementary material Table S2). This is most probably caused by infiltration of immune cells due to extensive brain tissue loss. Downregulated genes belonged to categories such as neuron development, differentiation and migration, chromatin assembly/organization and regulation of transcription (supplementary material Table S2).

Taken together, combined deletion of *Hdac1* and *Hdac2* resulted in severely impaired brain development. Deregulated patterns of histone acetylation and gene expression were accompanied by reduced proliferation and elevated DNA damage with subsequent activation of apoptosis in *Hdac1*^Δ/Δn^*Hdac2*^Δ/Δn^ mice. Collectively, this leads to the reduced size and impaired architecture of cortex, cerebellum and essentially the entire brain. Furthermore, we detected cerebral hemorrhage as a secondary effect due to dramatic tissue loss at E18.5. Our results indicate that HDAC1 and HDAC2 are indispensable for neural cell viability and brain development.

### A single *Hdac2* allele is sufficient for normal brain development

**Fig. 5:**
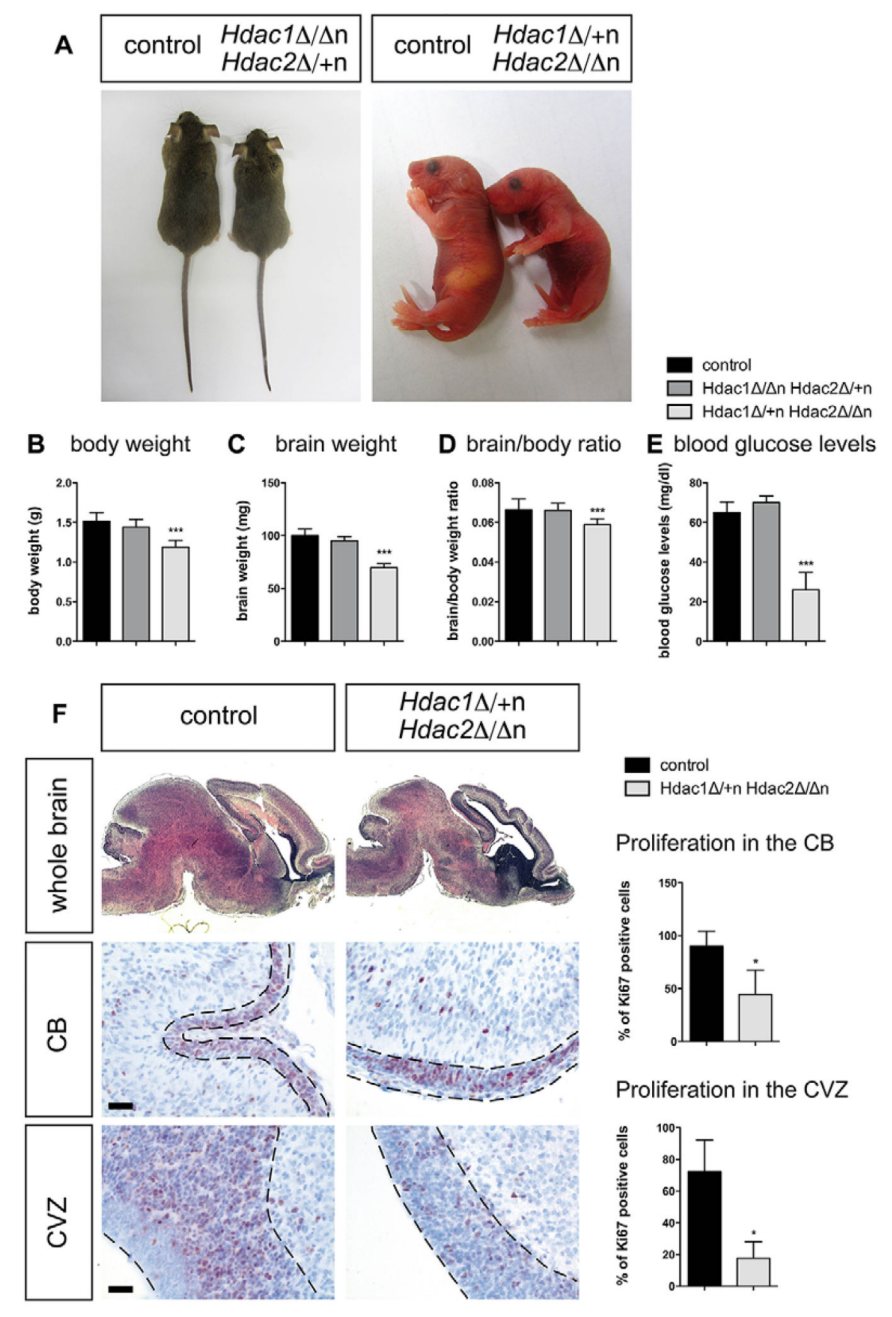
Divergent phenotypes upon deletion of different combinations of *Hdac1*/*Hdac2* alleles in the brain (A) Left panel: representative pictures of a wild-type (left) versus an *Hdac1*^Δ/Δn^*Hdac2*^Δ/+n^ (right) adult littermate. Right panel: representative pictures of a wild-type (left) versus an *Hdac1*^Δ/+n^*Hdac2*^Δ/Δn^ (right) newborn littermate. (B-D) Body/brain weights and ratios of P0 control (black, *n*=16) compared with *Hdac1*^Δ/Δn^*Hdac2*^Δ/+n^ (dark gray, *n*=5) and *Hdac1*^Δ/+n^*Hdac2*^Δ/Δn^ (light gray, *n*=16) mice. Error bars indicate s.d. ***P<0.001. (E) Blood glucose levels of P0 control (black, *n*=7) compared with *Hdac1*^Δ/Δn^*Hdac2*^Δ/+n^ (dark gray, *n*=5) and *Hdac1*^Δ/+n^*Hdac2*^Δ/Δn^ (light gray, *n*=7) mice. Error bars indicate s.d. ***P<0.001. (F) Whole brain: Hematoxylin and Eosin stainings on wild-type control littermates (left) and *Hdac1*^Δ/+n^*Hdac2*^Δ/Δn^ (right) paraffin sections. Detailed regions in the brain (cerebellum and cortical ventricular zone): IHC with the proliferation marker Ki67 antigen (brown staining) on wild-type littermates (left) and *Hdac1*^Δ/+n^*Hdac2*^Δ/Δn^ (right) paraffin sections. The nuclei are counterstained with Mayer’s hemalaun (blue staining). For quantification, positively stained cells in *Hdac1*^Δ/+n^*Hdac2*^Δ/Δn^ (light gray) and corresponding wild-type controls (black) were evaluated by the HistoQuest Software as shown in the graphs on the far right. Scale bars: 20 μm. Error bars indicate s.d. (*n*=3). *P<0.05. CB, cerebellum; CVZ, cortical ventricular zone.

Next, we asked whether expression of a single allele of either *Hdac1* or *Hdac2* could prevent embryonic lethality and brain abnormalities of *Hdac1*^Δ/Δn^*Hdac2*^Δ/Δn^ mice. Strikingly, mice with a single *Hdac2* allele (*Hdac1*^Δ/Δn^*Hdac2*^Δ/+n^) were viable and fertile, displayed normal brain development and exhibited no obvious phenotype except decreased body size and weight (Fig. 5A; supplementary material Fig. S6A). By contrast, *Hdac1*^Δ/+n^*Hdac2*^Δ/Δn^ mice revealed reduced body weight, brain size, brain/body weight ratio and blood glucose levels and died within few hours after birth (P0) (Fig. 5B-E). Brains of *Hdac1*^Δ/+n^*Hdac2*^Δ/Δn^ mice displayed smaller sizes of cerebellum and cortex and reduced foliation of the cerebellum (Fig. 5F). In accordance, *Hdac1*^Δ/+n^*Hdac2*^Δ/Δn^ brains showed diminished proliferation in cerebellum and cortical ventricular zone, whereas *Hdac1*^Δ/Δn^*Hdac2*^Δ/+n^ brains were indistinguishable from littermate controls (Fig. 5F; supplementary material Fig. S6B). In contrast to *Hdac1*^Δ/Δn^*Hdac2*^Δ/Δn^ mice, brains of *Hdac1*^Δ/+n^*Hdac2*^Δ/Δn^ and *Hdac1*^Δ/Δn^*Hdac2*^Δ/+n^ mice displayed no significant increase in DNA damage or apoptosis (supplementary material Fig. S3D-G).

In summary, expression of a single allele of either *Hdac1* or *Hdac2* prevented several pathologic features, including DNA damage, apoptosis, cerebral hemorrhage and the dramatic drop in total HDAC activity as observed in *Hdac1*^Δ/Δn^*Hdac2*^Δ/Δn^ mice. However, only expression of a single *Hdac2* allele was sufficient to entirely prevent the severe phenotype and embryonic lethality of *Hdac1*^Δ/Δn^*Hdac2*^Δ/Δn^ mice, whereas a single *Hdac1* allele delayed death to the perinatal period. Our findings highlight the predominant contribution of HDAC2 to brain development and survival.

### *Hdac1*^Δ/+n^*Hdac2*^Δ/Δn^ brains display decreased co-repressor-associated HDAC activity and deregulated gene expression

**Fig. 6:**
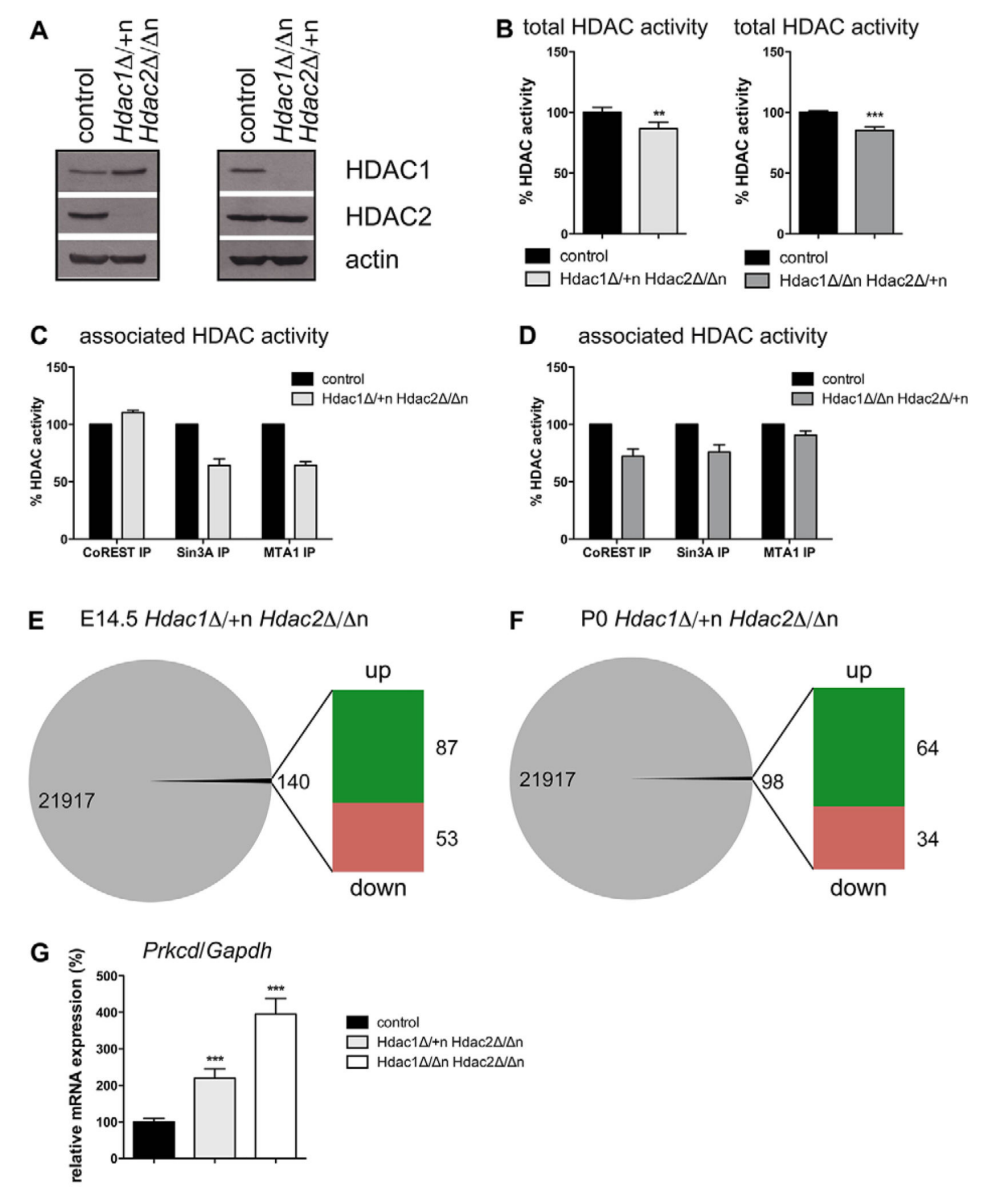
*Hdac1*^Δ/+n^*Hdac2*^Δ/Δn^ mice display reduced co-repressor complex activity and several deregulated target genes (A) Representative immunoblot analyses of P0 wild-type littermate controls versus *Hdac1*^Δ/+n^*Hdac2*^Δ/Δn^ (left panel) and *Hdac1*^Δ/Δn^*Hdac2*^Δ/+n^ (right panel) brain extracts. The membrane was probed with antibodies against HDAC1, HDAC2 and β-actin was used as loading control. (B) HDAC activities measured in P0 brain protein extracts from *Hdac1*^Δ/+n^*Hdac2*^Δ/Δn^ (left panel, light gray) and *Hdac1*^Δ/Δn^*Hdac2*^Δ/+n^ (right panel, dark gray) mice compared with wild-type littermate controls (black). Error bars indicate s.d. (*n*=4). **P<0.01; ***P<0.001. (C,D) For immunoprecipitations P0 brain protein extracts from *Hdac1*^Δ/+n^*Hdac2*^Δ/Δn^ (C) and *Hdac1*^Δ/Δn^*Hdac2*^Δ/+n^ (D) and the corresponding wild-type littermate controls were incubated with antibodies against CoREST, SIN3A and MTA1 and the associated HDAC activity was measured (*n*=2). The corresponding representative immunoblots are shown in supplementary material Fig. S8. (E,F) Agilent microarray gene expression analysis of *Hdac1*^Δ/+n^*Hdac2*^Δ/Δn^ and corresponding control mice at E14.5 (*n*=3) (E) and P0 (*n*=4) (F). 140 annotated genes at E14.5 (E) and 98 genes at P0 (F) brains were at least twofold deregulated (*P*<0.05). (G) Relative mRNA expression of *Prkcd* in E14.5 *Hdac1*^Δ/+n^*Hdac2*^Δ/Δn^ brains (light gray) and *Hdac1*^Δ/Δn^*Hdac2*^Δ/Δn^ brains (white) compared with the corresponding wild-type littermate controls (black). Values are normalized to the housekeeping gene *Gapdh*. Error bars indicate s.d. (*n*≥4). ***P<0.001.

To elucidate the mechanisms leading to the highly diverse phenotypes of *Hdac1*^Δ/+n^*Hdac2*^Δ/Δn^ and *Hdac1*^Δ/Δn^*Hdac2*^Δ/+n^ mice, we first examined HDAC1 and HDAC2 protein expression in newborn mouse brains. HDAC1 levels in *Hdac1*^Δ/+n^*Hdac2*^Δ/Δn^ brains were slightly elevated, whereas HDAC2 expression in *Hdac1*^Δ/Δn^*Hdac2*^Δ/+n^ brains was equivalent to wild-type littermates (Fig. 6A). Our results indicate operative functionality of the HDAC1/2 compensatory mechanism even if three of the four *Hdac1/2* alleles were ablated. Despite the difference in phenotypes, we observed a similar reduction in total cellular deacetylase activity in P0 brain extracts of *Hdac1*^Δ/+n^*Hdac2*^Δ/Δn^ and *Hdac1*^Δ/Δn^*Hdac2*^Δ/+n^ mice by 14% and 15%, respectively (Fig. 6B). Therefore, we compared histone acetylation patterns of *Hdac1*^Δ/+n^*Hdac2*^Δ/Δn^, *Hdac1*^Δ/Δn^*Hdac2*^Δ/+n^ and their respective littermate control brains. In contrast to *Hdac1*^Δ/Δn^*Hdac2*^Δ/Δn^, *Hdac1*^Δ/Δn^*Hdac2*^Δ/+n^ mice showed no differences in the abundance of specific histone acetylation marks (supplementary material Fig. S7B). Interestingly *Hdac1*^Δ/+n^*Hdac2*^Δ/Δn^ mice displayed a transient increase in H3K56 acetylation at E15.5 (supplementary material Fig. S7A), but not at P0 (supplementary material Fig. S7C).

To examine the association of HDAC1 and HDAC2 with co-repressor complexes we performed co-immunoprecipitation experiments for CoREST, SIN3A and MTA1 (NuRD). We observed a reduction of SIN3A- and NuRD-associated HDAC activity in *Hdac1*^Δ/+n^*Hdac2*^Δ/Δn^ mice, whereas in *Hdac1*^Δ/Δn^*Hdac2*^Δ/+n^ brains CoREST-associated HDAC activity was affected to a greater extent (Fig. 6C,D; supplementary material Fig. S8A,B). The reduced HDAC activity of NuRD and SIN3A complexes in *Hdac1*^Δ/+n^*Hdac2*^Δ/Δn^ brains could in part be explained by decreased MTA1 and SIN3A protein levels (supplementary material Fig. S8A). Given the divergent phenotypes of *Hdac1*^Δ/+n^*Hdac2*^Δ/Δn^ and *Hdac1*^Δ/Δn^*Hdac2*^Δ/+n^ mice, we explored brain-specific gene expression by microarray analysis. Strikingly, 140 genes (87 up, 53 down) were differentially expressed in *Hdac1*^Δ/+n^*Hdac2*^Δ/Δn^ brains, whereas only *Hdac1* was significantly deregulated in brains of *Hdac1*^Δ/Δn^*Hdac2*^Δ/+n^ mice at E14.5 (Fig. 6E; supplementary material Table S1). Based on the fact that *Hdac1*^Δ/+n^*Hdac2*^Δ/Δn^ mice die within the first day after birth, we additionally investigated differential gene expression on P0. Ninety-eight genes (64 up, 34 down) were significantly deregulated at P0 in *Hdac1*^Δ/+n^*Hdac2*^Δ/Δn^ mice (Fig. 6F; supplementary material Table S1). GO analysis revealed that proliferation and cell-cycle-associated genes were deregulated at E14.5, whereas genes encoding metabolic functions were commonly changed at P0 (supplementary material Table S2). These results confirmed the sufficiency of a single *Hdac2* allele to maintain wild-type gene expression levels and execute all essential functions of HDAC1 and HDAC2 in the embryonic mouse brain.

### PKCδ is a relevant target for HDAC1/HDAC2-mediated regulation of brain development

**Fig. 7:**
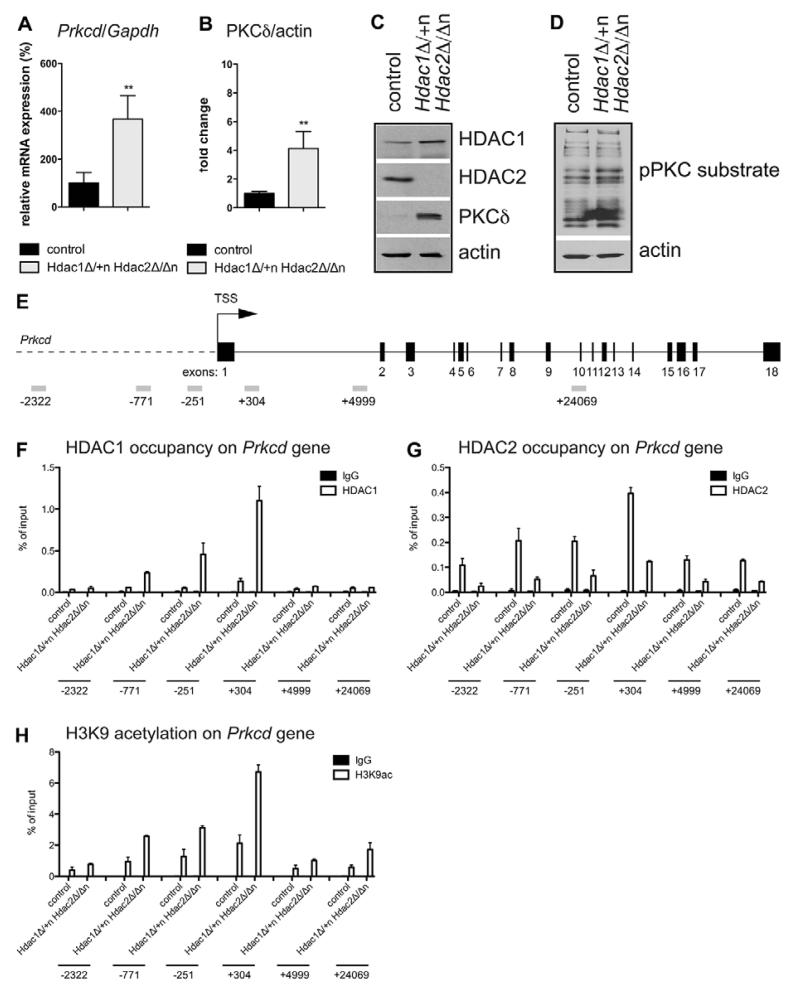
Upregulation of protein kinase C, delta in *Hdac1*^Δ/+n^*Hdac2*^Δ/Δn^ mice (A) Relative mRNA expression of *Prkcd* in P0 *Hdac1*^Δ/+n^*Hdac2*^Δ/Δn^ brains (light gray) compared with the corresponding wild-type littermate controls (black). Values are normalized to the housekeeping gene *Gapdh*. Error bars indicate s.d. (*n*=4). **P<0.01. (B) Quantification of PKCδ protein levels in P0 *Hdac1*^Δ/+n^*Hdac2*^Δ/Δn^ brains (light gray) compared with the corresponding wild-type littermate controls (black). Immunoblot signals were scanned using ImageQuant Software and values are normalized to β-actin. Error bars indicate s.d. (*n*=4). **P<0.01. (C) Immunoblot analysis of P0 wild-type littermate controls versus *Hdac1*^Δ/+n^*Hdac2*^Δ/Δn^ brain extracts with antibodies against HDAC1, HDAC2, PKCδ and β-actin as loading control. (D) Immunoblot analysis of P0 wild-type littermate control versus *Hdac1*^Δ/+n^*Hdac2*^Δ/Δn^ brain extracts with antibodies against pPKC substrate and β-actin as loading control. (E) Schematic representation of the *Prkcd* gene with exons depicted as numbered black boxes. Primers used for the chromatin-immunoprecipitation experiment are illustrated as gray rectangles. (F-H) Chromatin from littermate control and *Hdac1*^Δ/+n^*Hdac2*^Δ/Δn^ brains was immunoprecipitated with antibodies specific for HDAC1 (F), HDAC2 (G), H3K9ac (H) (white bars) and IgG as negative control (black bars) followed by quantitative reverse transcription polymerase chain reaction (qRT-PCR) with primers specific for different regions of the *Prkcd* gene as illustrated in E. Error bars indicate s.d. (*n*≥2). TSS, transcriptional start site.

Only five genes including downregulated *Hdac2* were commonly deregulated in *Hdac1*^Δ/+n^*Hdac2*^Δ/Δn^ brains at E14.5 and P0 (supplementary material Table S1). Interestingly, one of the identified genes (*Prkcd*) encodes protein kinase C delta (PKCδ). As PKCδ is an important regulator of proliferation, differentiation, apoptosis, autophagy and energy metabolism in mammalian cells (Kikkawa et al., 2002; Chen et al., 2009), we focused our analysis on this kinase. At E14.5 *Prkcd* expression was twofold induced in *Hdac1*^Δ/+n^*Hdac2*^Δ/Δn^ brains and fourfold in the absence of both enzymes (Fig. 6G). At P0 *Hdac1*^Δ/+n^*Hdac2*^Δ/Δn^ brains showed elevated levels of PKCδ mRNA and protein (fourfold) as well as increased enzymatic activity (Fig. 7A-D). Increased PKCδ expression was detected in several brain regions, including hypothalamus, hippocampus and regions around the rostral migratory stream (supplementary material Fig. S9A). Laser microdissection analysis revealed that *Prkcd* is upregulated mostly in neuron-rich areas of *Hdac1*^Δ/+n^*Hdac2*^Δ/Δn^ brains (supplementary material Fig. S10B). As PKCδ was also found upregulated in *Hdac1*^Δ/Δn^*Hdac2*^Δ/Δn^ brains (supplementary material Table S1), we assumed that the *Prkcd* gene is a direct target of HDAC1/HDAC2. To test this hypothesis we performed site-directed chromatin immunoprecipitation (ChIP) experiments with antibodies specific for HDAC1, HDAC2 and histone H3K9ac, a histone mark known to be a substrate for HDAC1/HDAC2 (Bhaskara et al., 2013) at different regions of the *Prkcd* gene locus (Fig. 7E). HDAC2 and to some extent HDAC1 were associated with regions surrounding exon 1 of the *Prkcd* gene in wild-type brains (Fig. 7F,G). Interestingly, in *Hdac1*^Δ/+n^*Hdac2*^Δ/Δn^ brains enhanced recruitment of HDAC1 was not sufficient to prevent an increase in local H3K9 acetylation and *Prkcd* mRNA expression, indicating that HDAC2 is the predominant regulator of the *Prkcd* gene (Fig. 7A,F-H). Given that HDAC2 is highly enriched in neurons (Fig. 1D; supplementary material Fig. S10C) and *Prkcd* was upregulated in neurons of *Hdac1*^Δ/+n^*Hdac2*^Δ/Δn^ brains (supplementary material Fig. S10A,B) we analyzed the recruitment of HDAC2 to *Prkcd* in neuron-specific ChIP assays. As expected, HDAC2 showed recruitment to the region around *Prkcd* exon 1 preferentially in neurons (supplementary material Fig. S10D). In summary, our data suggest that HDAC2 is required for the control of cell type-specific PKCδ levels in neurons.

**Fig. 8:**
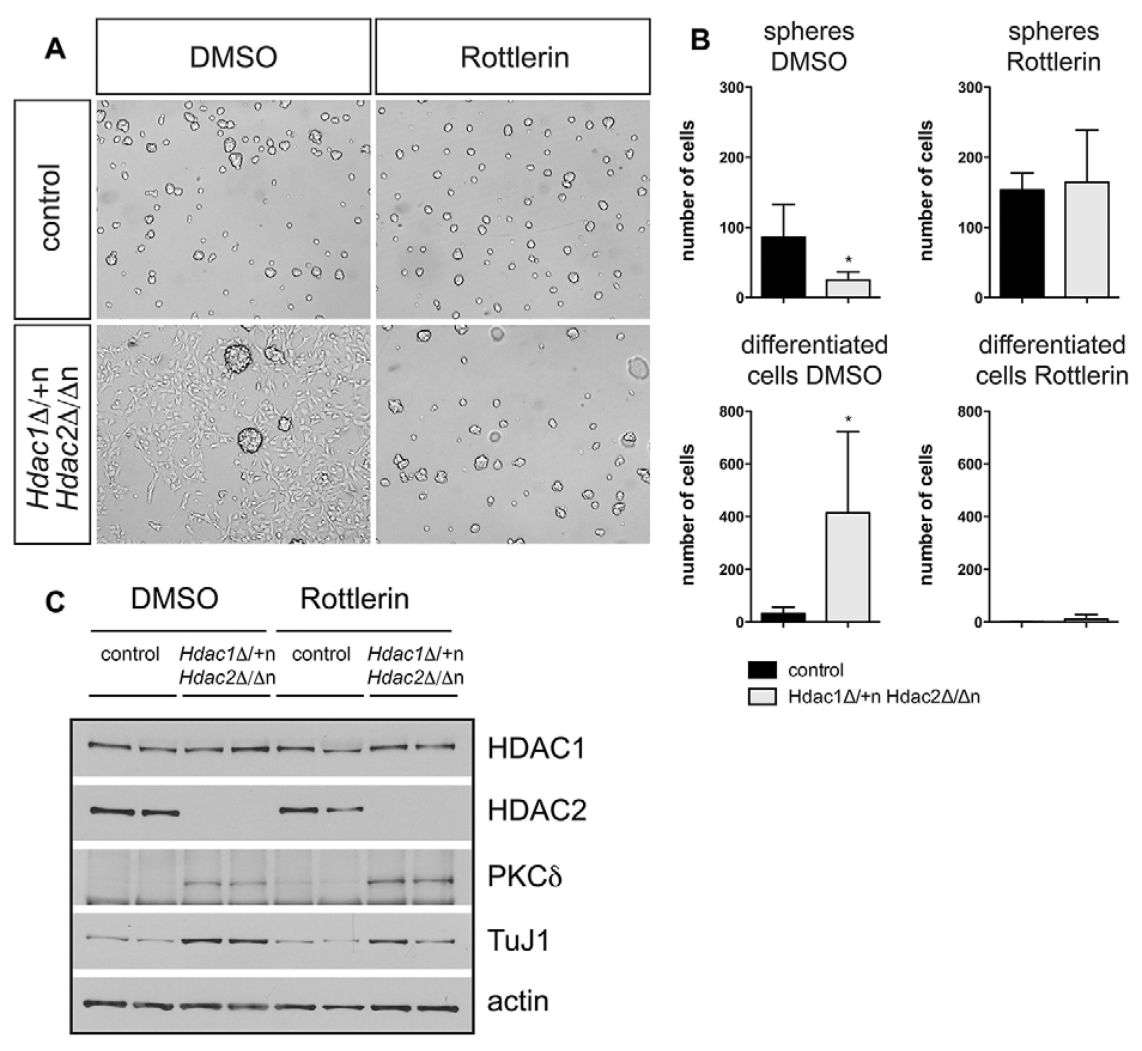
The PKC inhibitor Rottlerin reverts the phenotype of *Hdac1*^Δ/+n^*Hdac2*^Δ/Δn^ neurospheres (A) Representative pictures of control (upper panel) and *Hdac1*^Δ/+n^*Hdac2*^Δ/Δn^ (lower panel) *in vitro* neurospheres after treatment with DMSO (left panel) or 1 μM Rottlerin (right panel). (B) Quantification of spheres (upper panel) or differentiated cells (lower panel) for *Hdac1*^Δ/+n^*Hdac2*^Δ/Δn^ (light gray) and the corresponding wild-type littermate controls (black). Error bars indicate s.d. (*n*=4). *P<0.05. (C) Immunoblot analysis of wild-type control versus *Hdac1*^Δ/+n^*Hdac2*^Δ/Δn^ neurospheres treated with DMSO or Rottlerin. The membrane was probed with antibodies against HDAC1, HDAC2, TuJ1, PKCδ and β-actin as loading control.

Given that *Hdac1*^Δ/+n^*Hdac2*^Δ/Δn^ brains show disturbed development and reduced proliferation we performed *in vitro* neurosphere experiments to examine proliferation and differentiation of neural stem cells and progenitor cells derived from *Hdac1*^Δ/+n^*Hdac2*^Δ/Δn^ and littermate control brains. To test a potential impact of PKCδ on neural cell differentiation we treated neurosphere cultures with the PKC inhibitor Rottlerin. Compared with wild-type neurospheres, *Hdac1*^Δ/+n^*Hdac2*^Δ/Δn^ neurospheres expressed elevated levels of PKCδ, were formed with reduced efficiency and displayed a partially differentiated appearance with projections (Fig. 8A-C). High concentrations of Rottlerin (3 μM) led to impaired cell proliferation and cell death, suggesting a requirement for PKC activity during neurosphere proliferation (data not shown). Strikingly, treatment with moderate concentrations of Rottlerin (1 μM) rescued the phenotype of *Hdac1*^Δ/+n^*Hdac2*^Δ/Δn^ neurospheres. Accordingly, the spontaneous differentiation reflected by enhanced levels of the differentiation marker TuJ1 (neuron-specific class III β-tubulin; Tubb3 – Mouse Genome Informatics) was sensitive to Rottlerin treatment (Fig. 8A,C). Similarly, moderate shRNA-mediated knockdown of *Prkcd* partially rescued the spontaneous differentiation phenotype of *Hdac1*^Δ/+n^*Hdac2*^Δ/Δn^ (supplementary material Fig. S11). These data suggest that upregulation of PKCδ contributes to the brain developmental phenotype of *Hdac1*^Δ/+n^*Hdac2*^Δ/Δn^ mice.

## DISCUSSION

### Cell type-specific expression of HDAC1 and HDAC2

In this report we investigated the function of the class I deacetylases HDAC1 and HDAC2 during brain development. In contrast to most other organs and tissues, HDAC1 and HDAC2 showed cell type-specific expression patterns in the brain (MacDonald and Roskams, 2008; this study). Although both HDACs were expressed in neural progenitors, HDAC1 was preferentially expressed in astrocytes and HDAC2 was mainly expressed in adult neurons from P4 onwards. This cell type-specific expression pattern was not observed in *in vitro* astrocyte or neuron cultures isolated from embryonic brains indicating a requirement for stage-specific signals to control HDAC1 and HDAC2 expression patterns (data not shown). Analysis of *Hdac1* and *Hdac2* mRNA levels in astrocyte- or neuron-enriched brain regions suggested that the cell type-specific expression was mediated by translational or post-translational mechanisms.

Despite the restricted expression patterns in postnatal brains, single deletion of either *Hdac1* or *Hdac2* in neural cells had no obvious effect on general brain development as a result of compensation by the upregulated paralog. In accordance, a previous study had shown that ablation of *Hdac2* by GLAST::CreERT2 did not affect overall brain architecture, but led to aberrant maintenance of progenitor transcripts and defective neuronal maturation in adult neurogenesis (Jawerka et al., 2010). Similarly, *Nestin-Cre*-mediated deletion of *Hdac2* influenced neither brain anatomy nor cell positioning, but resulted in increased synapse number and memory enhancement (Guan et al., 2009). Moreover, HDAC2 has a unique role in synaptic transmission in mature neurons (Akhtar et al., 2009). Ablation of *Hdac1* or *Hdac2* in other cell types, including embryonic stem cells, fibroblasts, B cells, keratinocytes and various cell lines led to upregulation of the other enzyme (Lagger et al., 2002; Lagger et al., 2010; Yamaguchi et al., 2010; Chen et al., 2011; Jurkin et al., 2011), but to our knowledge this is the first study showing cross-regulation beyond the cell type.

### Combined deletion of *Hdac1* and *Hdac2* results in cellular lethality

By contrast, simultaneous ablation of both enzymes in neural stem cells and progenitors caused severe developmental abnormalities resulting in loss of most of the brain tissue at E18.5. Importantly, *Hdac1*^Δ/Δn^*Hdac2*^Δ/Δn^ brains displayed aberrant chromatin structures accompanied by increased histone acetylation levels, DNA damage and apoptosis. The effects caused by *Nestin-Cre*-mediated deletion of *Hdac1*/*Hdac2* were more dramatic than the reported phenotype of mice where *Hdac1* and *Hdac2* were ablated by *Gfap-Cre* (Montgomery et al., 2009). This might be due to different expression patterns of nestin and GFAP within neural stem cell and progenitor populations. Combined loss of HDAC1 and HDAC2 was shown to affect proliferation and differentiation in most cell types and tissues. For instance, simultaneous loss of HDAC1 and HDAC2 led to impaired oligodendrocyte development due to activation of the Wnt pathway (Ye et al., 2009) and strongly compromised Schwann cell myelination by affecting Sox10-dependent transcription (Jacob et al., 2011) and NF-κB activity (Chen et al., 2011). In other tissues, loss of HDAC1 and HDAC2 was linked to deregulation of the p53/p63 pathway in the epidermis (LeBoeuf et al., 2010), derepression of BMP4 and RB1 in the lung (Wang et al., 2013), defective T-cell receptor signaling in T cells (Dovey et al., 2013) or reduced autophagy in skeletal muscles (Moresi et al., 2012). Importantly, many cell types including B cells (Yamaguchi et al., 2010), transformed fibroblasts (Haberland et al., 2009), cardiomyocytes (Montgomery et al., 2007), Schwann cells (Jacob et al., 2011), oocytes (Ma et al., 2012), thymocytes (Heideman et al., 2013) and keratinocytes (Winter et al., 2013) showed increased apoptosis in the absence of HDAC1/HDAC2. We propose a model in which, in addition to transcriptional deregulation of important signaling pathways, a direct effect of HDAC1 and HDAC2 on the chromatin structure contributes to the lethality of HDAC1/HDAC2-deficient proliferating cells. Interestingly, HDAC1 and HDAC2 have been identified as components of the DNA damage response accountable for the removal of H3K56ac and H4K16ac (Miller et al., 2010) and pharmacological inhibition of both enzymes has been shown to result in replication stress and DNA damage (Bhaskara et al., 2013). Recently, SIRT1 was found to deacetylate and thereby activate HDAC1 to maintain genomic stability in neurons *in vitro* (Dobbin et al., 2013). It is conceivable that, in the absence of HDAC1 and HDAC2, increased levels of specific acetylation marks such as H3K56ac and H4K16ac in *Hdac1*^Δ/Δn^*Hdac2*^Δ/Δn^ brains lead to aberrations in chromatin structures and consequently in DNA damage and apoptosis. In conclusion, HDAC1 and HDAC2 might be required to preserve a normal chromatin structure in addition to their transcriptional regulator function. According to this model, complete loss of HDAC1 and HDAC2 is not compatible with cell proliferation, indicating that drugs inhibiting the activities of both enzymes have the potential of anti-tumor agents.

### Haploinsufficiency of *Hdac1* in the absence of HDAC2

The DNA damage/apoptosis phenotype of HDAC1/HDAC2-deficient brains was prevented by a single allele of either *Hdac1* or *Hdac2*, suggesting that both enzymes have the capacity to fulfill the vital cellular functions required in neural cells. However, *Hdac1*^Δ/Δn^*Hdac2*^Δ/+n^ mice showed normal brain development and no obvious phenotype, whereas *Hdac1*^Δ/+n^*Hdac2*^Δ/Δn^ mice displayed impaired brain development with reduced proliferation and increased differentiation. This was not due to differences in the overall cellular HDAC activities in the brains of mutant mice but is more likely to be caused by differences in co-repressor complex activities resulting in changes in gene expression profiles selectively in *Hdac1*^Δ/+n^*Hdac2*^Δ/Δn^ brains. Reduced activity of SIN3A and NuRD co-repressor complexes in *Hdac1*^Δ/+n^*Hdac2*^Δ/Δn^ brains was accompanied by decreased protein levels of SIN3A and MTA1. A similar reduction in MTA1 and SIN3A expression was observed upon deletion of two *Hdac1* alleles and one *Hdac2* allele in T cells (Dovey et al., 2013) and epidermis (Winter et al., 2013), suggesting a potential scaffolding function of HDAC1/HDAC2. The cause for perinatal lethality of *Hdac1*^Δ/+n^*Hdac2*^Δ/Δn^ mice is presently unclear; however, it is conceivable that compromised brain development or peripheral nervous system defects influence mobility, coordination, olfactory recognition and/or suckling behavior of newborn mice.

Interestingly, the opposite effect, haploinsufficiency of *Hdac2* in the absence of HDAC1 in the epidermis, resulted in strongly impaired epidermal development (Winter et al., 2013). In this case, mobilization of epidermal stem cells led to hyperproliferation and increased differentiation. A similar positive effect on cell proliferation by a single *Hdac2* allele in the absence of HDAC1 in T cells was shown to favor tumor formation (Dovey et al., 2013; Heideman et al., 2013). By contrast, a more important function of HDAC2 was found in oocytes, where this enzyme regulates chromosome segregation and kinetochore function (Ma and Schultz, 2013). In summary, these data indicate overlapping but specific functions of HDAC1 and HDAC2 during mouse development.

### PKCδ overexpression contributes to the phenotype of *Hdac1*^Δ/+n^*Hdac2*^Δ/Δn^ mice

One relevant target gene commonly deregulated in E14.5 and P0 *Hdac1*^Δ/+n^*Hdac2*^Δ/Δn^ mice encodes the serine/threonine kinase PKCδ. This enzyme mediates diverse signal transduction pathways regulating proliferation, differentiation, apoptosis and autophagy and is expressed in a variety of tissues and cell types, including the nervous system (Miyamoto et al., 2002; Carracedo et al., 2013). Overexpression or activation of PKCδ has been shown to suppress proliferation in different cell lines (Watanabe et al., 1992; Mischak et al., 1993; Mandil et al., 2001; Cerda et al., 2006) and primary cells (Fukumoto et al., 1997; Harrington et al., 1997; Miyamoto et al., 2002; Chew et al., 2011). Depending on the cell type, PKCδ signaling can also promote differentiation (Deucher et al., 2002; di Giacomo et al., 2010; Nitti et al., 2010; Park and Patel, 2010; Hamdorf et al., 2011; Feng et al., 2012).

In addition to modulating the proliferation/differentiation balance, PKCδ signaling is also implicated in regulating apoptosis and autophagy (Chen et al., 2009; Jin et al., 2011). As absence of HDAC1/HDAC2 in skeletal muscles was recently shown to cause a defect in autophagy flux and a concomitant increase in the LC3 marker (Moresi et al., 2012), we monitored autophagy in *Hdac1*^Δ/+n^*Hdac2*^Δ/Δn^ brains by LC3 immunoblots, but found no change in autophagy flux compared with control littermates (supplementary material Fig. S9B). Similarly, *Hdac1*^Δ/+n^*Hdac2*^Δ/Δn^ brains did not exhibit increased apoptosis. However, *Hdac1*^Δ/+n^*Hdac2*^Δ/Δn^ brains and neurospheres displayed reduced proliferation. Moreover, *Hdac1*^Δ/+n^*Hdac2*^Δ/Δn^ neurospheres showed a more differentiated appearance and upregulation of the neuronal differentiation marker TuJ1. This proliferation/differentiation shift presumably occurred due to PKCδ overexpression, as neurosphere treatment with the PKC inhibitor Rottlerin or shRNA-mediated knockdown of *Prkcd* reverted the spontaneous differentiation phenotype and restored wild-type proliferation. We propose that overexpression of PKCδ in *Hdac1*^Δ/+n^*Hdac2*^Δ/Δn^ brains leads to premature differentiation of neural progenitors resulting in reduced proliferation and impaired brain development.

In summary, we have shown that simultaneous loss of HDAC1 and HDAC2 in neural cells results in aberrant chromatin structures, DNA damage and apoptosis, indicating a crucial but redundant role of these enzymes in chromatin organization. Expression of single alleles of *Hdac1* or *Hdac2* in the absence of its paralog revealed a major role of HDAC2 during brain development and survival.

## MATERIALS AND METHODS

### Animal care and transgenic mouse lines

All experiments were performed in accordance with the Austrian guideline for animal care and protection. All mouse lines were bred to a mixed genetic background of C57BL/6J × 129SV. To delete *Hdac1*/*Hdac2* alleles in the nervous system mice with floxed *Hdac1*/*Hdac2* alleles (Yamaguchi et al., 2010) were mated with *Nestin-Cre* mice (Tronche et al., 1999).

### shRNA-mediated silencing

For gene silencing pLKO.1 lentiviral vectors with shRNA expression cassettes targeting mouse *Prkcd* and corresponding controls were generated and used for infection of primary mouse neurospheres (without polybrene) as previously described (Lagger et al., 2010). Following transduction, cells were selected with 2 mg/ml puromycin.

### EdU incorporation

Gravid mice were injected intraperitoneally with 300 μl 5-ethynyl-2′-deoxyuridine (EdU; Invitrogen) diluted in DMSO (2.5 mg/ml, 10 mM). Mice were sacrificed 1 hour after EdU injection and E14.5 embryos were dissected. The embryonic brain tissue was homogenized and used for the Click-iT EdU Flow Cytometry Assay Kit (Molecular Probes) according to the manufacturer’s protocol. Proliferation profile and intracellular stain were analyzed on FACS Aria (BD Biosciences).

### Blood glucose measurements

Blood glucose levels of newborn mice were determined with test strips and the OneTouch UltraEasy glucose meter (LifeScan).

### RNA isolation and qRT-PCR analysis

Brains were isolated and homogenized in TRIzol reagent (Invitrogen). Total RNA was isolated following the manufacturer’s instructions. RNA was reversely transcribed with the iScript cDNA synthesis kit (Bio-Rad). Real time PCR analysis was performed with the KAPA SYBR FAST qPCR MasterMix (Peqlab) on an iCycler IQ system (Bio-Rad). Data were normalized to the housekeeping gene *Gapdh*.

### Microarray and data processing

Analysis of gene expression data was performed using the Bioconductor software (www.bioconductor.org) (Gentleman et al., 2004) and the script written in R. Raw intensities were imported into Bioconductor and further processed with the limma (Smyth, 2004) package. Normalization between arrays was performed using the quantile method, duplicate probes were averaged and a linear model was fitted with contrasts for knockout/wild-type effects. Cut-offs for differential expression were set to a minimum twofold up- or downregulation and a maximum adjusted *P*-value of 0.05.

### ChIP and PCR analysis

Isolated brains were finely chopped, washed with PBS and crosslinked with disuccinimidyl glutarate (DSG) (2 mM, AppliChem) for 25 minutes at room temperature. After another PBS washing step the brains were cross-linked by adding formaldehyde (to a final concentration of 1%) at room temperature for 10 minutes. The cross-linking process was stopped by addition of glycine to a final concentration of 125 mM. Chromatin isolation procedure was followed as previously described (Hauser et al., 2002). For ChIP equal amounts of sonicated chromatin were diluted tenfold and precipitated overnight with the following antibodies: HDAC1 (Sat13, Seiser lab), HDAC2 (Bethyl Laboratories), H3K9ac (Millipore), C-terminal H3 (clone 1B1-B2, Active Motif) and rabbit IgG (Invitrogen) as a control. Chromatin antibody complexes were isolated using protein A-beads for rabbit primary antibodies or for G-beads for mouse primary antibodies (Dynabeads, Invitrogen). The PCRs with 1:20 dilutions of genomic DNA (input) were carried out together with the precipitated DNA. The extracted DNA was used for quantitative PCR analysis with the primers listed in supplementary material Table S3.

For neuron-specific ChIP mice carrying an enhanced green fluorescent protein (EGFP)-tagged version of wild-type MECP2 (Lyst et al., 2013) were used for FACS sorting of neuronal brain nuclei and consecutive ChIP analysis. Nuclei from MECP2-EGFP brains were isolated as previously described (Klose and Bird, 2004) and stored in resuspension buffer [20% glycerol, sodium butyrate, protease inhibitor cocktail (Roche) in PBS] at −80°C until further use. On the day of FACS sorting, nuclei were equilibrated in PBS containing 2 mM DSG and incubated at room temperature for 15 minutes. After a washing step in PBS, a second cross-linking step with 1% formaldehyde was performed for 8 minutes at room temperature followed by the addition of glycine to a final concentration of 125 mM. Cross-linked nuclei were washed and resuspended in PBTB Buffer (0.1% Triton X-100, 5% BSA in PBS). Nuclei were sorted on a FACS Aria according to intensity of GFP expression into GFP-high (neuronal nuclei) and GFP-low (non-neuronal nuclei) populations. Sorted nuclei were centrifuged at 3500 ***g*** for 10 minutes at 4°C and resuspended in lysis buffer. ChIP was performed as described above.

### Protein isolation, immunoblot analysis and HDAC activity assays

Dissected brains were immediately frozen in liquid nitrogen and stored at −80°C. For protein extraction, frozen brains were manually homogenized in HUNT buffer (20 mM Tris-HCl pH 8.0, 100 mM sodium chloride, 1 mM EDTA, 0.5% NP-40) supplemented with protease inhibitor cocktail (Roche) and phenylmethanesulfonyl fluoride (PMSF) in a glass homogenizer. After full speed centrifugation, the supernatant containing the soluble protein fraction was further used. Equal amounts of 20-30 μg of proteins were separated by sodium dodecyl sulfate-polyacrylamide gel electrophoresis (SDS-PAGE) (10% gels) and transferred onto nitrocellulose membranes (Protran, Whatman) according to standard protocols. For detection the Enhanced Chemiluminescence Kit (PerkinElmer) was used. HDAC activity assays were performed with brain protein extracts as previously described (Lagger et al., 2002). Primary antibodies for immunoblotting: HDAC1 (10E2 or Sat13), HDAC2 (3F3), SIN3A (sc-994, Santa Cruz), CoREST (07-455, Millipore), MTA1 (sc-9446, Santa Cruz), PKCδ (610397, BD), TuJ1 (ab14545, Abcam), phospho-(Ser) PKC Substrate (2261, Cell Signaling), LC3 (0260-100, NanoTools), β-actin (A5316, Sigma).

### Co-immunoprecipitation assay

Total protein extracts from brain were harvested as described above. Equal amounts of 1 mg of protein were incubated for 1 hour at 4°C with 4 μg antibody. The immunoprecipitation was carried out using protein A-beads or protein G-beads (Dynabeads^©^, Invitrogen) overnight at 4°C. The immune complexes were washed three times with HUNT buffer. Samples were used for an HDAC activity assay or they were heated in SDS sample buffer and used for immunoblotting. Primary antibodies used for co-immunoprecipitation: SIN3A (sc-994X, Santa Cruz), CoREST (07-455, Millipore), MTA1 (sc-9446 Santa Cruz).

### Histone immunoblot analysis

Dissected brains were immediately frozen in liquid nitrogen and stored at −80°C. Frozen brains were manually homogenized in lysis buffer (10 mM Tris-HCl pH 6.5, 50 mM sodium disulfite, 10 mM MgCl_2_, 10 mM sodium butyrate, 8.6% sucrose, 1% Triton X-100) supplemented with Protease inhibitor cocktail (Roche) and PMSF. Histone isolation was performed as previously described (Taplick et al., 1998). Equal amounts of histones (2 μg) were separated by SDS-PAGE and transferred onto nitrocellulose membranes (Protran, Whatman) according to standard protocols. The following antibodies were used: H3 C-terminal (ab1791, Abcam), H3K14ac (07-353, Millipore), H3K27ac (ab4729, Abcam), H3K4ac (39381, Active Motif), H3K56ac (ab76307, Abcam), H3K9ac (06-942, Millipore), H4K12ac (Sat44, Seiser Lab), H4K16ac (Sat53, Seiser Lab), H4K8ac (Sat198, Seiser Lab).

### Histological and IHC analyses

Tissue sample were fixed overnight in 4% paraformaldehyde and further embedded in paraffin. All stainings were performed on 4 μm sections. Hematoxylin and Eosin (H&E) stainings were carried out according to the standard procedure with an ASS1 staining unit (Pathisto). Fluorescence stainings were performed with the DyLight System (ThermoScientific) or the Tyramide Signal Amplification Kit (PerkinElmer), according to the manufacturer’s instruction. The slides were counterstained with DAPI and mounted in ProLong Gold (Invitrogen). PKCδ stainings were performed on 12 μm cryosections, which were air-dried, washed in PBS, blocked with 1% bovine serum albumin (BSA), 0.1% Triton X-100 in PBS and incubated in the primary PKCδ antibody (610397, BD) overnight followed by washing and incubation with the secondary antibody (DyLight 488; ThermoScientific).

Ki67 IHC detection was done with the IDetect Super Stain System HRP (ID laboratories), visualized with 3-amino-9-ethylcarbazole (ID laboratories) and counterstained with Hematoxylin.

Primary antibodies used for IHC: Ki67 (NovoCastra), HDAC1 (ab7028, Abcam), HDAC2 (3F3), NeuN (MAB377C3, Chemicon), GFAP (3670, Cell Signaling), H3K56ac (ab76307, Abcam), γH2AX (phospho S139) (ab2893, Abcam), cleaved caspase-3 (9661, Cell Signaling), TuJ1 (ab14545, Abcam).

### Microscopy

IHC stainings and whole embryos were imaged on a Zeiss stereomicroscope with camera. IHC fluorescence stainings were captured on a LSM Meta 510 (Zeiss) confocal microscope.

### Laser microdissection

Dissected brains were immediately frozen in liquid nitrogen, embedded in optimal cutting temperature compound (OCT; Tissue Tek) and 12 μm cryosections were acetone-fixed on PET membranes (Leica). Slides were dried, rinsed, stained with Cresyl Violet, washed and laser dissection was performed on an LMD6500-Laser Capture Microdissection/Imaging Unit (Leica). Regions enriched for astrocytes and neurons were dissected and used for RNA isolation with the Purelink Micro Kit (Invitrogen) according to the manufacturer’s protocol.

### Neurosphere assay

The neurosphere assay was modified from Deleyrolle and Reynolds (Deleyrolle and Reynolds, 2009). Whole E14.5 brains were dissected and mechanically dissociated until the suspension was homogenous. After centrifugation, the pellet was resuspended in an appropriate amount of neuronal proliferation medium containing neurobasal medium (Invitrogen), glutamine (Invitrogen), glutamax (Invitrogen), B27 (Invitrogen), 20 ng/μl recombinant human EGF (Cell Signaling), 20 ng/μl recombinant human basic FGF (Cell Signaling) and 5 μg/μl heparin (Sigma). After 4 days, cells were gently dissociated with 0.05% trypsin/EDTA (Invitrogen) and seeded for treatments. Twenty-four hours after seeding, neurospheres were treated for 48 hours either with 1 μM Rottlerin (Zhang et al., 2007) or as a negative control with the solvent dimethyl sulfoxide (DMSO) only. After treatment, cells were either pelleted and frozen for immunoblot analysis or plated for additional 24 hours on poly-L-lysine/laminin coated glass coverslips for microscopy.

### Statistical analysis

Real-time PCR and ChIP experiments were evaluated with Microsoft Excel. Relative intensities of bands detected in immunoblots were estimated using the ImageQuant software and relative protein expression levels were normalized to β-actin or the signal of H3 C-terminal antibody. For quantification of the Ki67 staining the HistoQuest software (TissueGnostics) was used. The significance between groups was determined by the unpaired Student’s *t*-test. *P*-values were calculated with the Graph-Pad Prism software and standard deviation (s.d.) is shown. *P<0.05; **P<0.01; ***P<0.001.

## Supplementary Material

SUPPLEMENTARY INFORMATION

Table S1

Table S2

Table S3

## Author contributions

A.H., S.L., O.P. and C. Seiser conceived and designed the experiments. A.H., S.L., J.K., A.L., M.A., O.P., J.Z., S.W., Y.X., M.S. and C. Schöfer performed the experiments. A.H., S.L., O.P., J.A.K., H.L. and C. Schöfer analyzed the data. G.B., P.M. and J.S. contributed transgenic mice/reagents/materials. A.H., S.L. and C. Seiser wrote the paper.
