# Supplementary material for: A single allele of Hdac2 but not Hdac1 is sufficient for normal mouse brain development in the absence of its paralog
Source: Development. Author manuscript; Available in PMC 2016 Mar 2. (PMC4773893; doi:10.1242/dev.100487)
Supplement: SUPPLEMENTARY INFORMATION [file NIHMS66893-supplement-SUPPLEMENTARY_INFORMATION.pdf]

SUPPLEMENTARY INFORMATION

A single allele of *Hdac2* but not *Hdac1* is sufficient for normal mouse brain development in the absence of its paralog

Astrid Hagelkruys, Sabine Lagger, Julia Krahmer, Alexandra Leopoldi, Matthias Artaker, Oliver Pusch, Jürgen Zezula, Simon Weissmann, Yunli Xie, Christian Schöfer, Michaela Schlederer, Gerald Brosch, Patrick Matthias, Jim Selfridge, Hans Lassmann, Jürgen A. Knoblich and Christian Seiser

SUPPLEMENTARY FIGURES (Figs. S1 - S11)

SUPPLEMENTARY TABLES (provided as Excel files: Supplementary Tables S1, S2 and S3)

SUPPLEMENTARY FIGURES

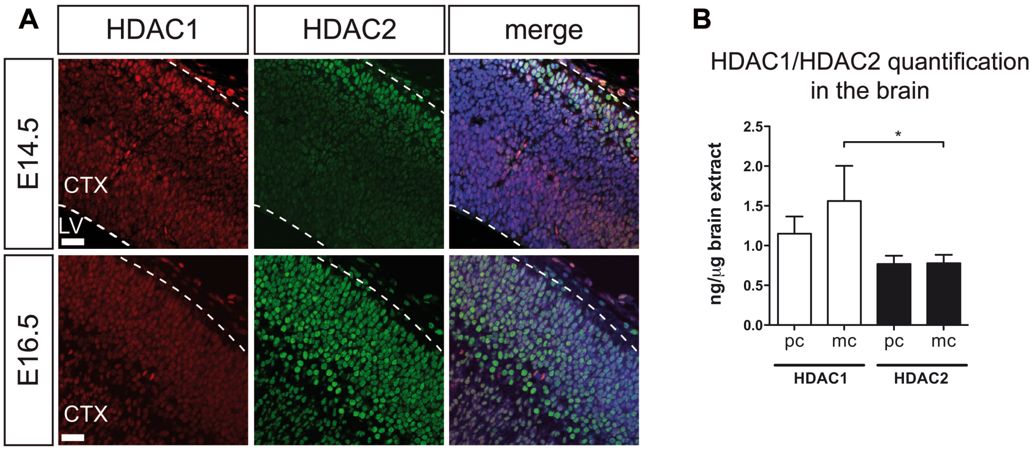

Figure S1. HDAC1/HDAC2 expression in the wild-type brain.
(A) Representative fluorescent IHC stainings of HDAC1 (red) and HDAC2 (green) in E14.5 (upper panel) and E16.5 (lower panel) wild-type mice. Nuclei are counterstained with DAPI. Scale bar: 20 µm. CTX, cortex; LV, lateral ventricle. **(B)** Quantification of HDAC1 (white) and HDAC2 (black) protein levels in wild-type P0 brain extracts by calibration of monoclonal (mc) and polyclonal (pc) antibodies with recombinant proteins. Immunoblot signals were detected and quantified with the Odyssey detection system. Monoclonal antibodies: HDAC1 (10E2, Seiser Lab), HDAC2 (3F3, Seiser Lab), polyclonal antibodies: HDAC1 (Sat13, Seiser Lab), HDAC2 (ab7029, *Abcam*). Error bars indicate s.d. (n=3). *P<0.05.

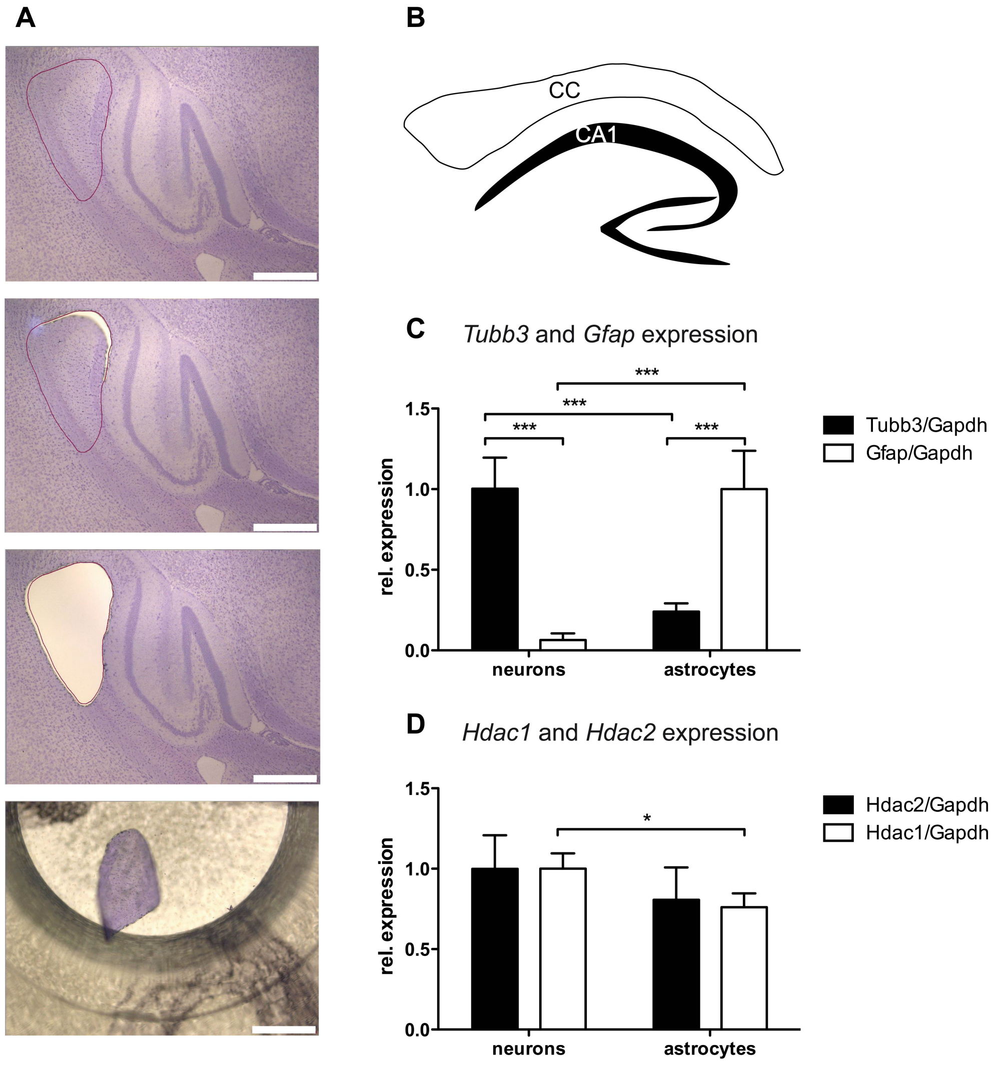

Figure S2. Similar mRNA levels for *Hdac1* and *Hdac2* in astrocytes and neurons.
(A) Procedure of laser microdissection of adult Cresyl violet stained cryosections. As highlighted, in this case parts of the corpus callosum were selected, dissected and used for RNA isolation and subsequent expression analysis. Scale bar: 400 µm. **(B)** Scheme of the brain regions enriched in astrocytes (CC, corpus callosum; white) and densely packed with neurons (CA1, hippocampal CA1 region; black), which were also used for laser microdissection. **(C)** Relative mRNA expression of *Tubb3* (black) and *Gfap* (white) in dissected neuron- and astrocyte-enriched brain regions as shown in **(B)**. Values are normalized to the housekeeping gene *Gapdh*. Error bars indicate s.d. (n=4). ***P<0.001. **(D)** Relative mRNA expression of *Hdac2* (black) and *Hdac1* (white) in dissected neuron- and astrocyte-enriched brain regions as shown in **(B)**. Values are normalized to the housekeeping gene *Gapdh*. Error bars indicate s.d. (n=3). *P<0.05.

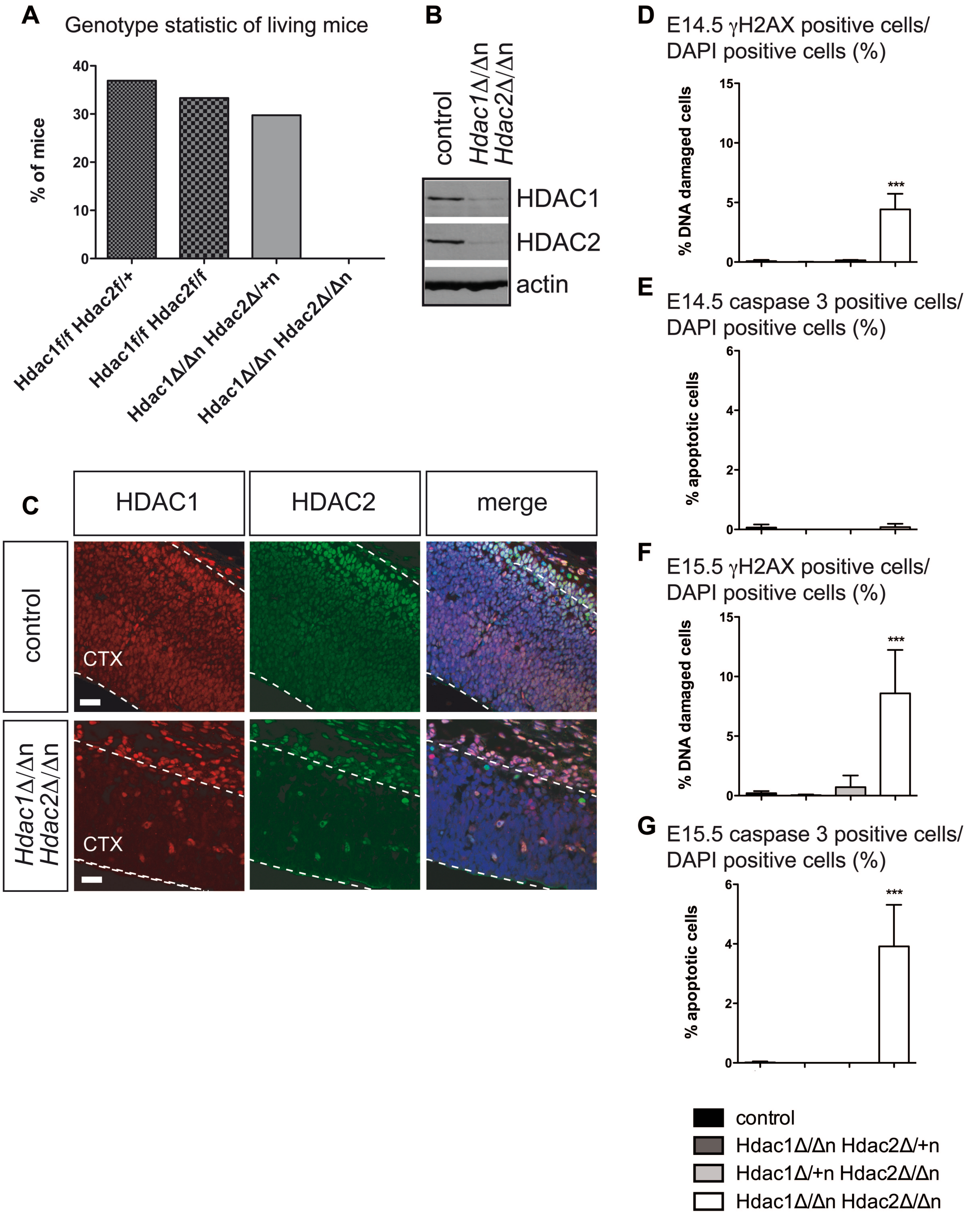

Figure S3. DNA damage, subsequent apoptosis and death prior birth occur specifically in *Hdac1*$^{\Delta/\Delta n}$*Hdac2*$^{\Delta/\Delta n}$ mice.

(A) Percentage of genotypes of living mice. Each of the four genotypes should occur with a probability of 25%. In total 84 mice were analyzed. **(B)** Representative immunoblot analysis of E14.5 wild-type littermate controls versus *Hdac1*$^{\Delta/\Delta n}$*Hdac2*$^{\Delta/\Delta n}$ brain extracts. The membrane was probed with antibodies against HDAC1, HDAC2 and β-actin was used as loading control. **(C)** Representative fluorescent IHC stainings of HDAC1 (red) and HDAC2 (green) in E14.5 *Hdac1*$^{\Delta/\Delta n}$*Hdac2*$^{\Delta/\Delta n}$ (lower panel) and wild-type littermate control (upper panel) mice. Nuclei are counterstained with DAPI. Scale bar: 20 µm. CTX, cortex. **(D,F)** Quantification of γH2AX positively stained cells in *Hdac1*$^{\Delta/\Delta n}$*Hdac2*$^{\Delta/+n}$ (dark gray), *Hdac1*$^{\Delta/+n}$*Hdac2*$^{\Delta/\Delta n}$ (light gray), *Hdac1*$^{\Delta/\Delta n}$*Hdac2*$^{\Delta/\Delta n}$ (white) and the corresponding wild-type control mice (black) at E14.5 **(D)** and E15.5 **(F)**. Error bars indicate s.d. (n=5 for control, n=2 for *Hdac1*$^{\Delta/\Delta n}$*Hdac2*$^{\Delta/+n}$, *Hdac1*$^{\Delta/+n}$*Hdac2*$^{\Delta/\Delta n}$ and *Hdac1*$^{\Delta/\Delta n}$*Hdac2*$^{\Delta/\Delta n}$). ***$P<0.001$. **(E,G)** Quantification of cleaved caspase 3 positively stained cells in *Hdac1*$^{\Delta/+n}$*Hdac2*$^{\Delta/\Delta n}$ (light gray), *Hdac1*$^{\Delta/\Delta n}$*Hdac2*$^{\Delta/\Delta n}$ (white) and the corresponding wild-type control mice (black) at E14.5 **(E)** and E15.5 **(G)**. Error bars indicate s.d. (n=5 for control, n=2 for *Hdac1*$^{\Delta/\Delta n}$*Hdac2*$^{\Delta/+n}$, *Hdac1*$^{\Delta/+n}$*Hdac2*$^{\Delta/\Delta n}$ and *Hdac1*$^{\Delta/\Delta n}$*Hdac2*$^{\Delta/\Delta n}$). ***$P<0.001$.

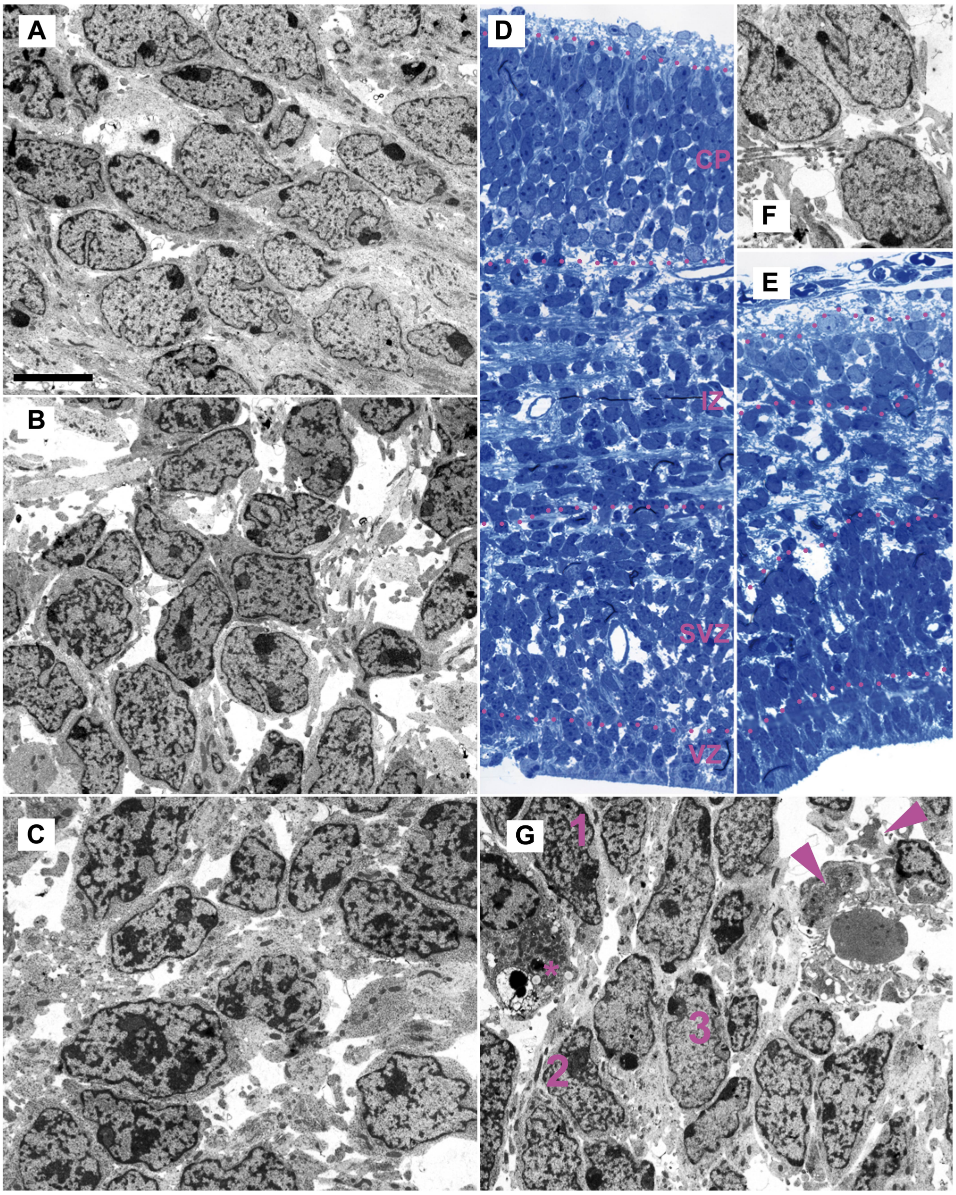

Figure S4. Ultrastructure of E15.5 wild-type and *Hdac1*$^{\Delta/\Delta n}$*Hdac2*$^{\Delta/\Delta n}$ cortices.
Electron micrographs **(A-C)** and semi-thin section **(D)** of wild-type control mice show typical well-separated layering **(D)**. The ultrastructure of nuclei from cortical plate **(A)**, intermediate zone **(B)**, and sub-ventricular zone **(C)** display different patterns of heterochromatin distribution, i.e. coarse reticulate in **(C)**, fine reticulate in **(B)**, and fine-disperse with large chromocenters at the nuclear periphery in **(A)**. In contrast, in *Hdac1*$^{\Delta/\Delta n}$*Hdac2*$^{\Delta/\Delta n}$ mice much less cells reach the cortical plate (**E**; semi-thin section); those cells that reach the cortical plate show normal nuclear ultrastructure (compare **(F)** to **(A)**). Notably however, the sub-ventricular zone of *Hdac1*$^{\Delta/\Delta n}$*Hdac2*$^{\Delta/\Delta n}$ mice consists of a mixture of cells, many of which display the nuclear morphology of more outer layers **(G)**; 1, nucleus resembling nuclei in sub-ventricular plate in the wild-type littermate control **(C)**; 2, nucleus resembling nuclei of intermediate zone in the wild-type littermate control **(B)**; 3, nucleus resembling nuclei of cortical plate in the wild-type control **(A)**. Additionally, cell debris (arrowheads) can be seen as well as cells displaying phagocytosed particles (asterisk). Scale bar: 5 µm. VZ, ventricular zone; SVZ, sub-ventricular zone; IZ, intermediate zone; CP, cortical plate.

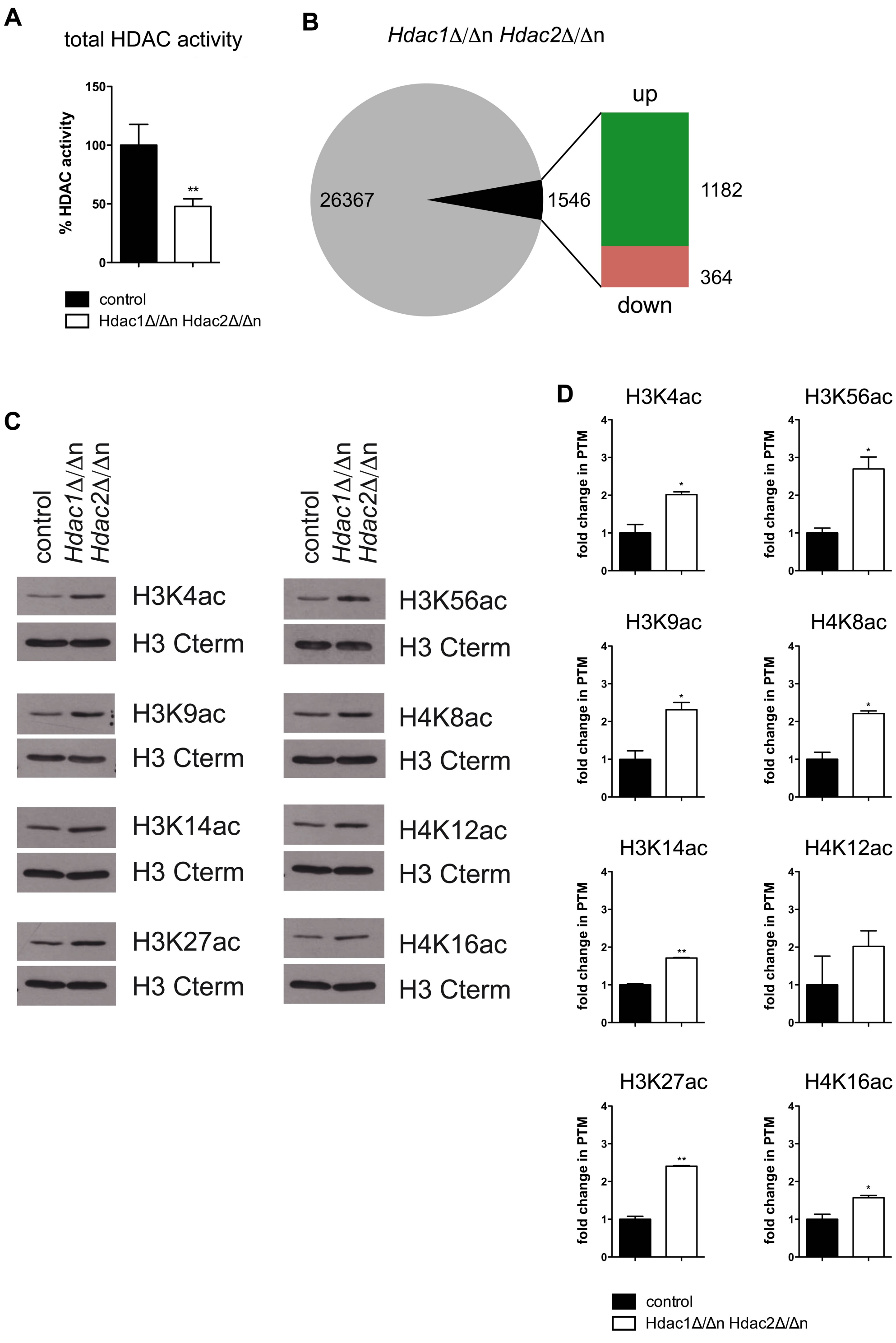

Figure S5. *Hdac1*$^{\Delta/\Delta n}$*Hdac2*$^{\Delta/\Delta n}$ brains display reduced total HDAC activity and increased histone acetylation marks.

(A) HDAC activities measured in E14.5 brain protein extracts from *Hdac1*$^{\Delta/\Delta n}$*Hdac2*$^{\Delta/\Delta n}$ mice (white) compared to wild-type littermate controls (black). Error bars indicate s.d. (n=3). **P<0.01. **(B)** Agilent microarray gene expression analysis of *Hdac1*$^{\Delta/\Delta n}$*Hdac2*$^{\Delta/\Delta n}$ and control mice at E14.5 (n=3). 1546 annotated genes were > twofold deregulated (p<0.05). **(C)** Histone blot analyses of E15.5 wild-type littermate controls versus *Hdac1*$^{\Delta/\Delta n}$*Hdac2*$^{\Delta/\Delta n}$ brain histone extracts. The membranes were probed with antibodies against H3K4ac, H3K9ac, H3K14ac, H3K27ac, H3K56ac, H4K8ac, H4K12ac, H4K16ac and for each blot the H3 C-terminal antibody was used as loading control. **(D)** Quantification of histone acetylation levels in E15.5 *Hdac1*$^{\Delta/\Delta n}$*Hdac2*$^{\Delta/\Delta n}$ brains (white) compared to wild-type littermate controls (black). Histone blot bands were scanned using ImageQuant Software. Error bars indicate s.d. (n=2). *P<0.05; **P<0.01.

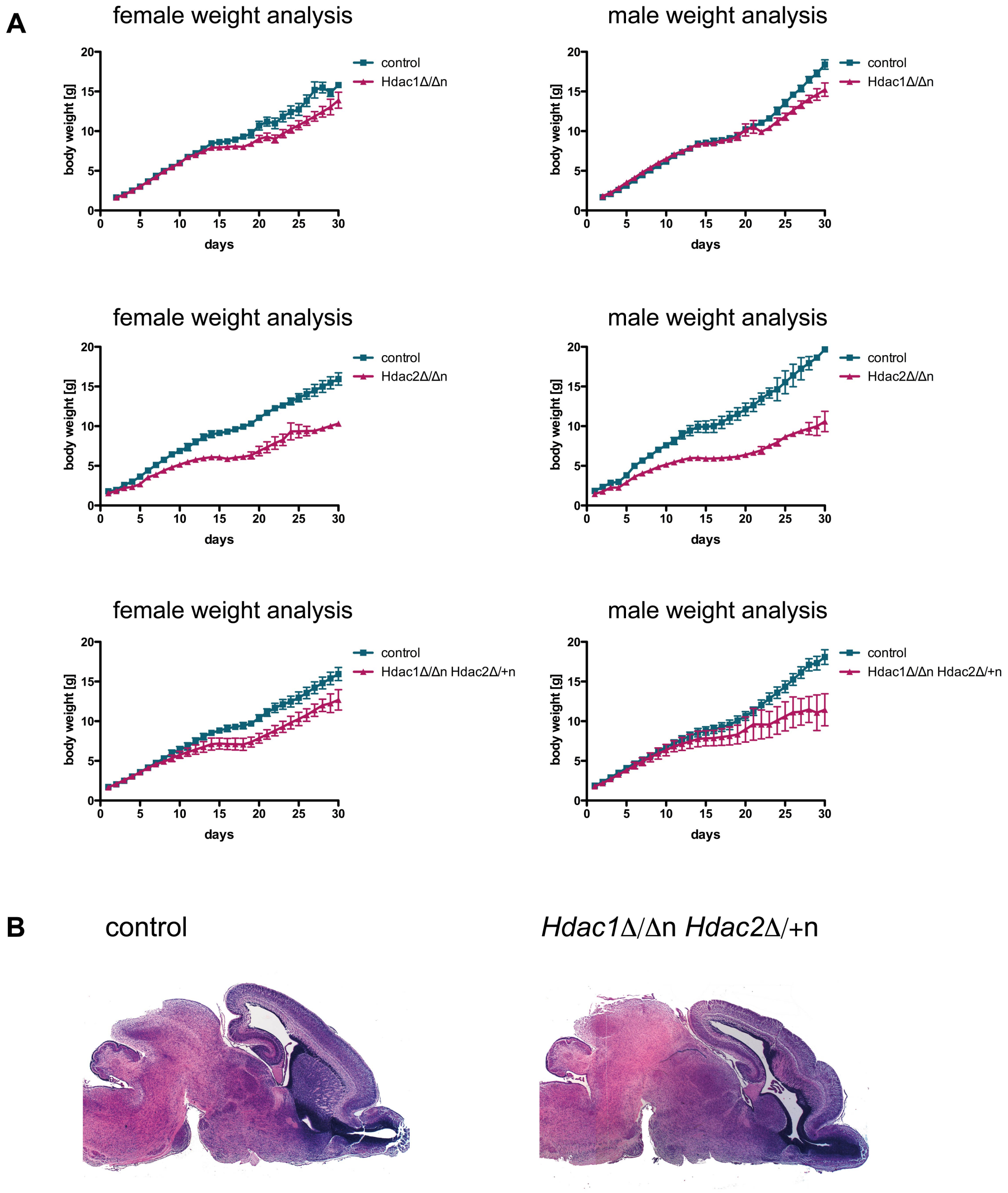

Figure S6. Diverse combinations of *Hdac1* and *Hdac2* alleles have a different impact on the body weight and on brain architecture. (A) Body weight of *Hdac1*$^{\Delta/\Delta n}$ (upper panel), *Hdac2*$^{\Delta/\Delta n}$ (middle panel) and *Hdac1*$^{\Delta/\Delta n}$*Hdac2*$^{\Delta/+n}$ (lower panel) mice compared to wild-type littermates in their first 30 days (n≥3). For *Hdac1*$^{\Delta/+n}$*Hdac2*$^{\Delta/\Delta n}$ there is no weight curve, since those mice die at P0. Graphs of female mice are on the left and male weight curves on the right. Weights of the different knockout mice are depicted in pink and their control littermates in blue. **(B)** Brain Hematoxylin/Eosin stainings on wild-type control littermates (left) and *Hdac1*$^{\Delta/\Delta n}$*Hdac2*$^{\Delta/+n}$ (right) paraffin sections.

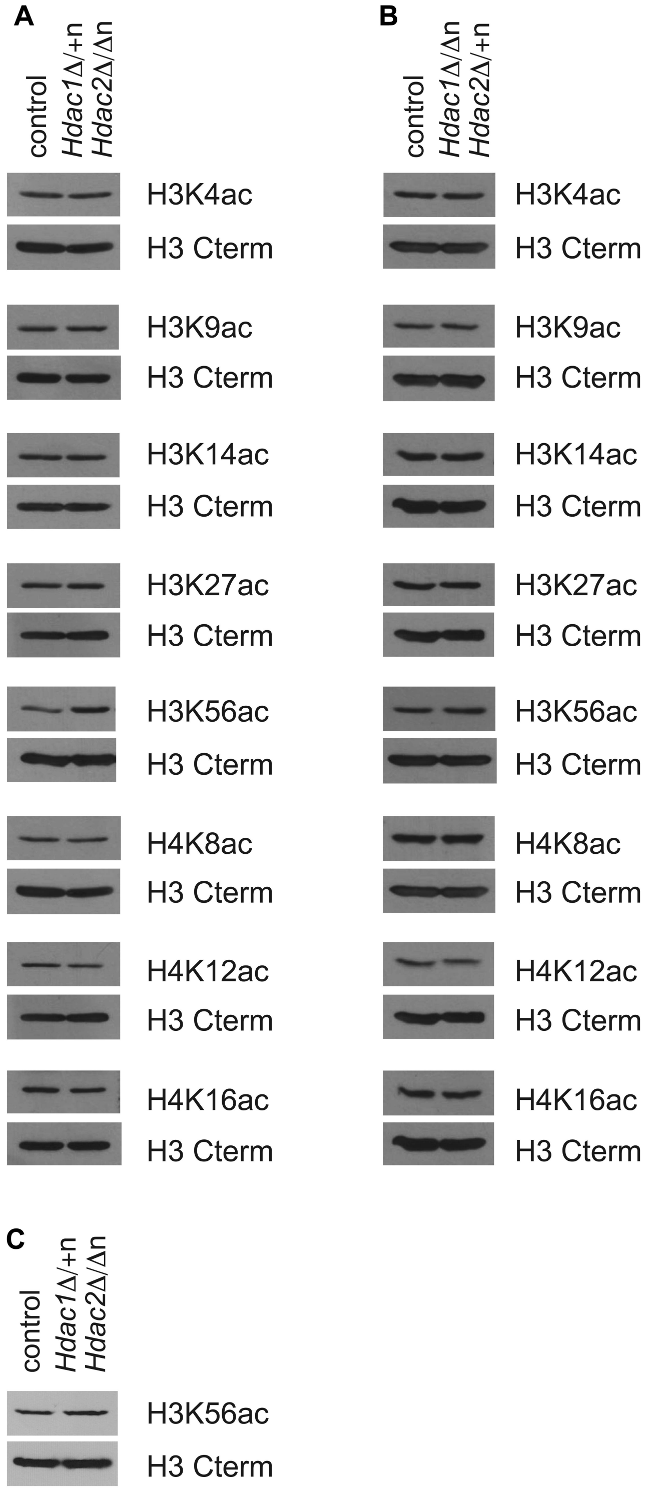

Figure S7. Histone acetylation patterns of *Hdac1*$^{\Delta/+n}$*Hdac2*$^{\Delta/\Delta n}$ and *Hdac1*$^{\Delta/\Delta n}$*Hdac2*$^{\Delta/+n}$ brains.

(A-B) Histone blot analyses of E15.5 wild-type littermate controls versus *Hdac1*$^{\Delta/+n}$*Hdac2*$^{\Delta/\Delta n}$ **(A)** and *Hdac1*$^{\Delta/\Delta n}$*Hdac2*$^{\Delta/+n}$ **(B)** brain histone extracts. The membranes were probed with antibodies against H3K4ac, H3K9ac, H3K14ac, H3K27ac, H3K56ac, H4K8ac, H4K12ac, H4K16ac and for each blot the H3 C-terminal antibody was used as loading control. **(C)** Histone blot analyses of P0 wild-type littermate controls versus *Hdac1*$^{\Delta/+n}$*Hdac2*$^{\Delta/\Delta n}$ brain histone extracts. The membrane was probed with an antibody against H3K56ac and the H3 C-terminal antibody was used as loading control.

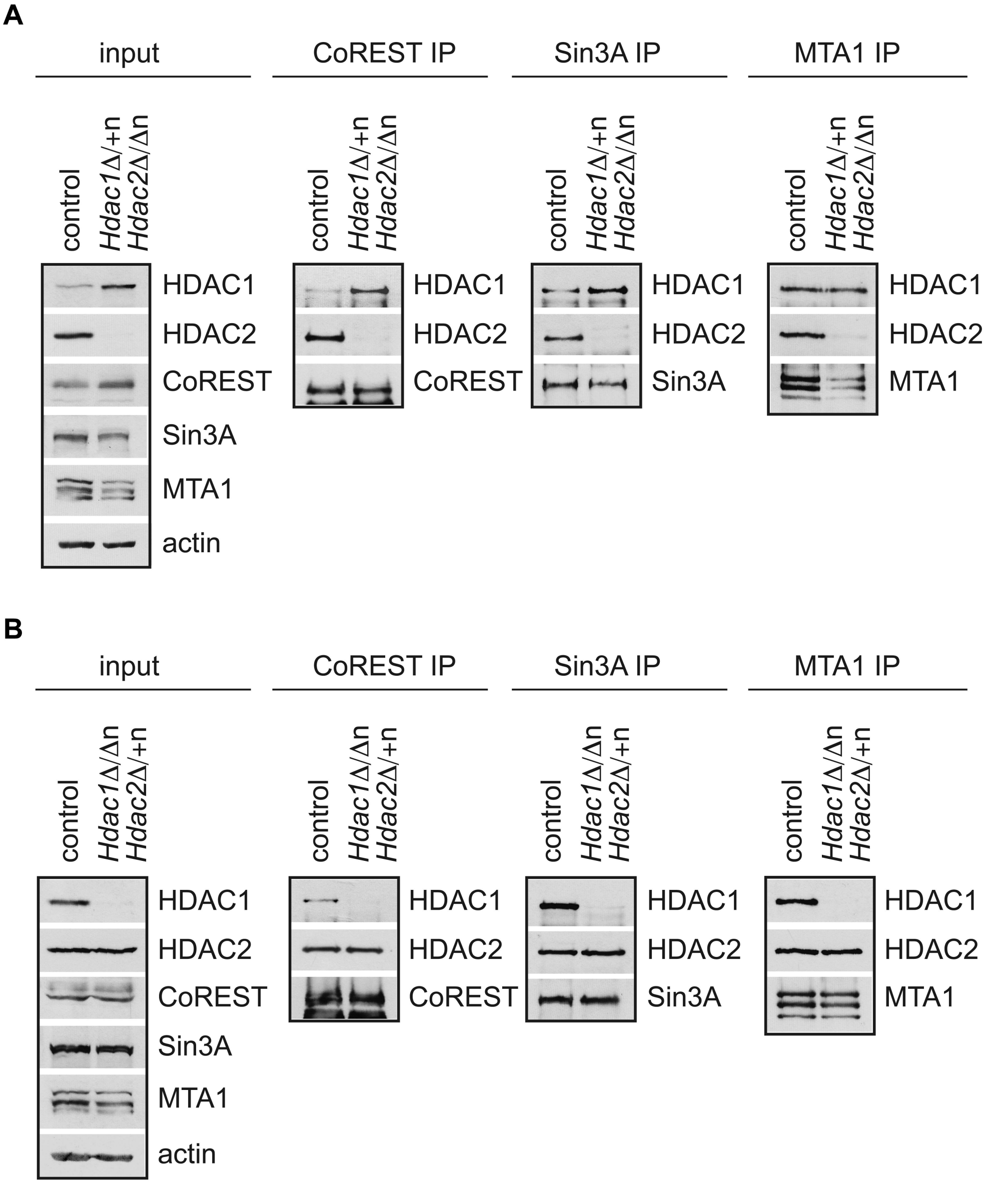

Figure S8. Association of HDAC1 and HDAC2 with co-repressor complexes in *Hdac1*$^{\Delta/+n}$ *Hdac2*$^{\Delta/\Delta n}$ and *Hdac1*$^{\Delta/\Delta n}$ *Hdac2*$^{\Delta/+n}$ brains.

For immunoprecipitations P0 brain protein extracts from *Hdac1*$^{\Delta/+n}$*Hdac2*$^{\Delta/\Delta n}$ **(A)** and *Hdac1*$^{\Delta/\Delta n}$*Hdac2*$^{\Delta/+n}$ **(B)** and the corresponding wild-type littermate controls were incubated with antibodies against CoREST, SIN3A and MTA1 and immunoblot analyses were performed with inputs and IPs. The membranes were probed with antibodies against HDAC1, HDAC2, CoREST, SIN3A, MTA1 and β-actin was used as loading control.

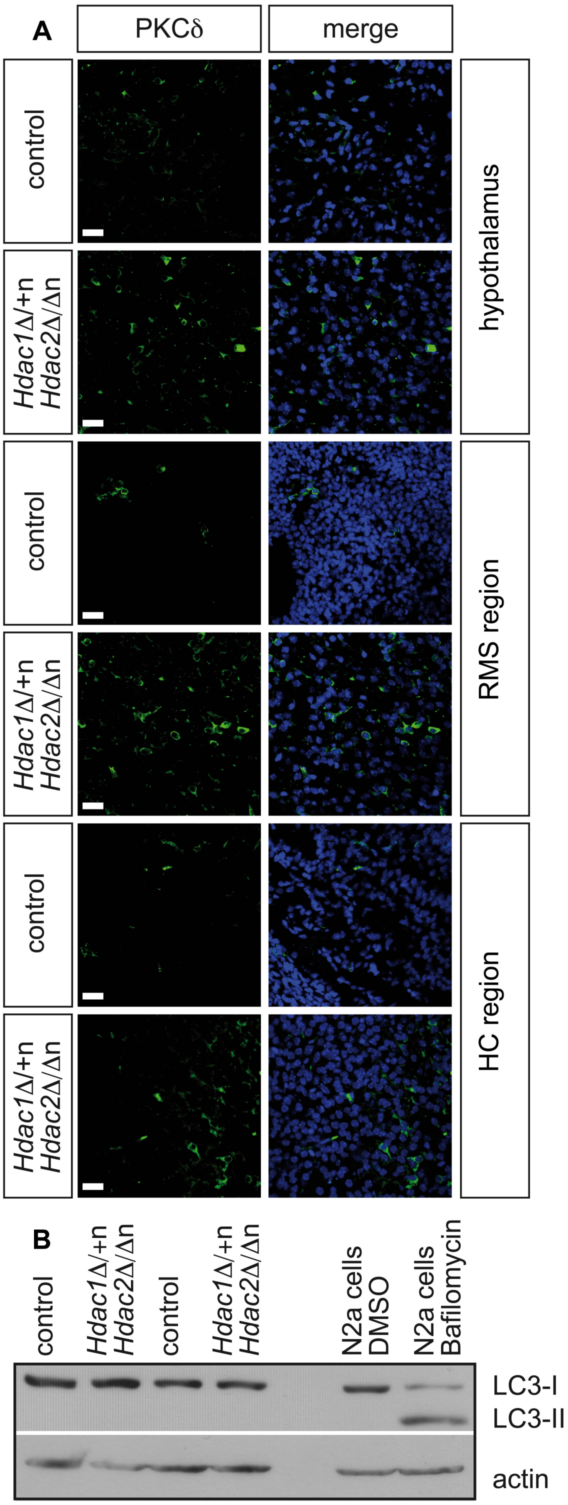

Figure S9. PKCδ is overexpressed in different regions of the *Hdac1*$^{\Delta/+n}$*Hdac2*$^{\Delta/\Delta n}$ brain, but does not lead to increased autophagic marker LC3.

(A) Fluorescent cryo-IHC stainings of PKCδ (green) in different regions of P0 wild-type versus *Hdac1*$^{\Delta/+n}$*Hdac2*$^{\Delta/\Delta n}$ brains. Nuclei are counterstained with DAPI. Scale bar: 20 μm. RMS, rostral migratory stream; HC, hippocampus. **(B)** Immunoblot analysis of two P0 wild-type littermate control versus two *Hdac1*$^{\Delta/+n}$*Hdac2*$^{\Delta/\Delta n}$ brain extracts. The membrane was probed with the LC3 antibody and β-actin was used as loading control. The LC3 antibody recognizes both forms of LC3: the cytoplasmic LC3-I (18 kDa) as well as the smaller lipidated form LC3-II (16 kDa) generated during autophagosome formation. As a positive control for LC3-II, N2a cells were treated with 10 μg/ml Bafilomycin A1 for 18 hours, which disturbed the fusion between autophagosomes and lysosomes and thereby led to accumulation of autophagosomes. As a control, N2a cells were treated with DMSO alone.

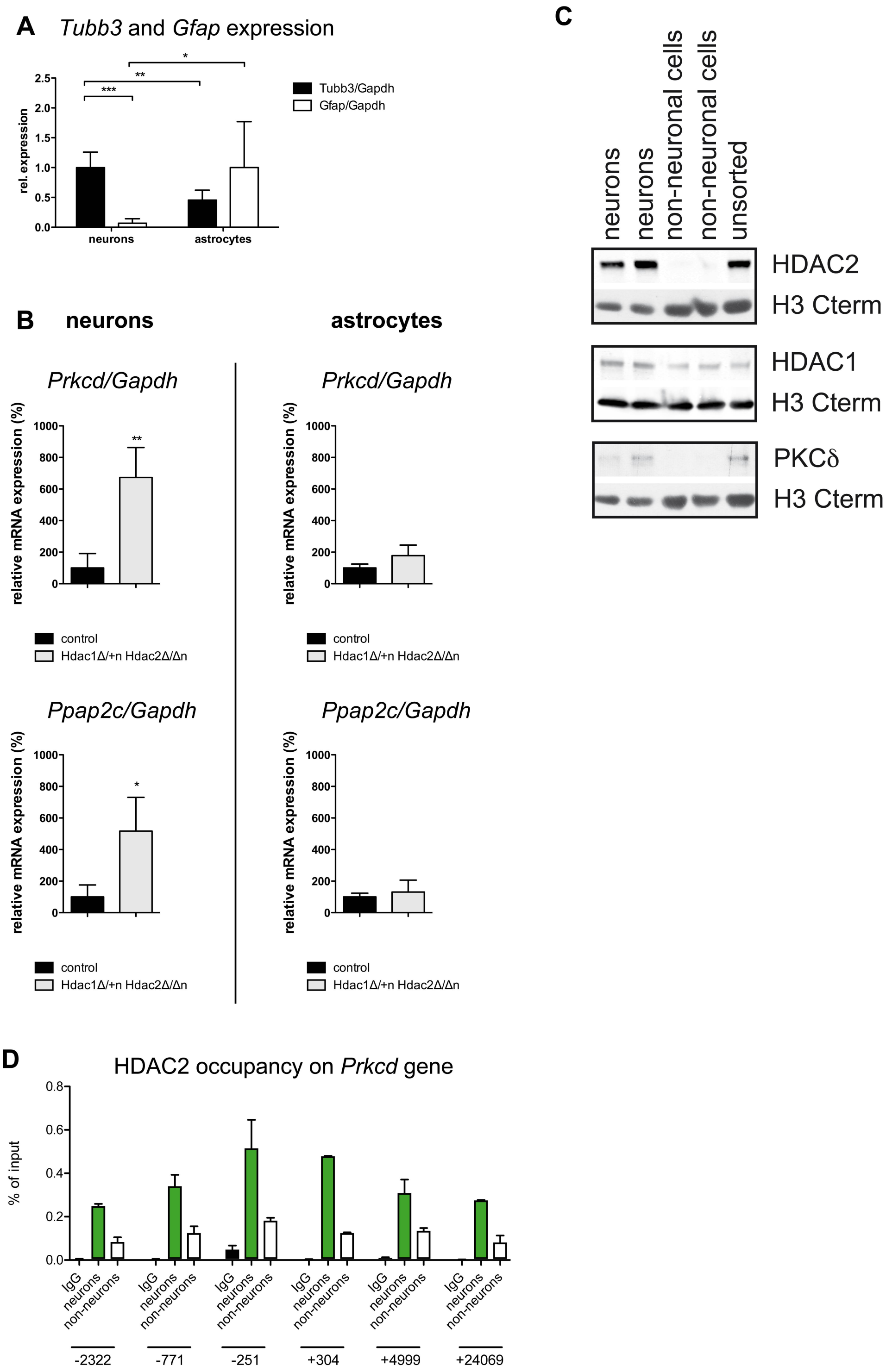

Figure S10. Deregulated target genes are specifically overexpressed in *Hdac1*$^{\Delta/+n}$*Hdac2*$^{\Delta/\Delta n}$ neurons.

(A) Relative mRNA expression of *Tubb3* (black) and *Gfap* (white) in dissected neuron- and astrocyte-enriched brain regions at P0. Values are normalized to the housekeeping gene *Gapdh*. Error bars indicate s.d. (n=6). *P<0.05; **P<0.01; ***P<0.001. Despite the fact that the laser microdissection was not as efficient as in the adult brain (shown in Fig. S2C), a substantial enrichment for neurons was achieved. **(B)** Relative mRNA expression of *Prkcd* (upper panel) and *Ppap2c* (lower panel) in dissected neuron- (left) and astrocyte-enriched (right) brain regions of *Hdac1*$^{\Delta/+n}$*Hdac2*$^{\Delta/\Delta n}$ brains (light gray) compared to their wild-type littermates (black). Values are normalized to the housekeeping gene *Gapdh*. Error bars indicate s.d. (n=3). *P<0.05; **P<0.01. **(C)** Immunoblot analyses with FACS-sorted nuclei of neurons and non-neuronal cells of adult wild-type brains expressing MECP2-EGFP. The membranes were probed with antibodies against HDAC2, HDAC1, PKCδ and the H3 C-

terminal antibody was used as loading control. **(D)** ChIP analysis of chromatin isolated from neuronal (green bars) and non-neuronal (white bars) nuclei. Chromatin was immunoprecipitated with antibodies specific for HDAC2 and IgG as negative control (black bars) followed by qRT-PCR with primers specific for different regions of the *Prkcd* gene as illustrated in Fig. 7E.

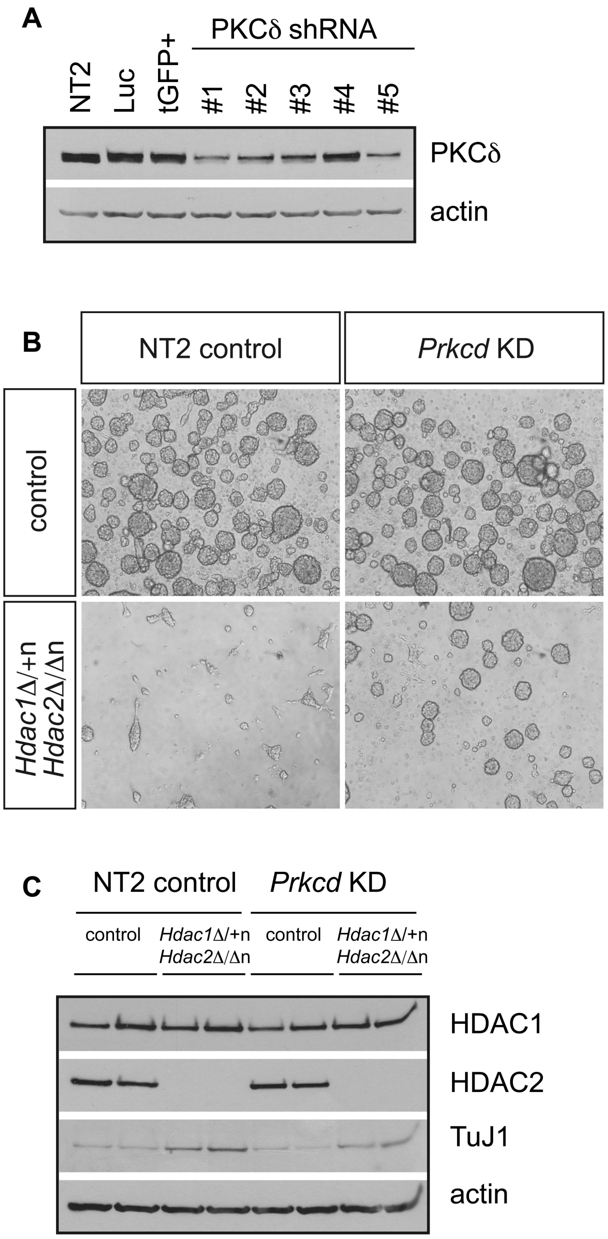

Figure S11. Knockdown of *Prkcd* attenuates the phenotype of *Hdac1*$^{\Delta/+n}$*Hdac2*$^{\Delta/\Delta n}$ neurospheres. (A) Immunoblot analysis of the knockdown efficiency of 5 different shRNAs targeting mouse *Prkcd* (#1-5) in comparison to non-target control shRNA NT2 by lentiviral infection of mouse N2a cells. Other controls: Luc, Luciferase shRNA; tGFP+, TurboGFP. The membrane was probed with antibodies against PKCδ and β-actin was used as loading control. KD, knockdown. **(B)** Representative pictures of control (upper panel) and *Hdac1*$^{\Delta/+n}$*Hdac2*$^{\Delta/\Delta n}$ (lower panel) *in vitro* neurospheres after expression of the non-target control shRNA NT2 (left panel) and the shRNA #2 targeting *Prkcd* (right panel) by lentiviral infection. **(C)** Immunoblot analysis of wild-type control versus *Hdac1*$^{\Delta/+n}$*Hdac2*$^{\Delta/\Delta n}$ neurospheres expressing the non-target control shRNA NT2 or the shRNA #2 targeting *Prkcd*. The membrane was probed with antibodies against HDAC1, HDAC2, TuJ1 and β-actin was used as loading control. KD, knockdown.

SUPPLEMENTARY TABLES

Table S1. Deregulated genes in the brain of E14.5 and P0 *Hdac1*$^{\Delta/\Delta n}$*Hdac2*$^{\Delta/+n}$ (HD2s), *Hdac1*$^{\Delta/+n}$*Hdac2*$^{\Delta/\Delta n}$ (HD1s) and *Hdac1*$^{\Delta/\Delta n}$*Hdac2*$^{\Delta/\Delta n}$ (DKO) mice ($p<0.05$, at least twofold change in expression). Up-regulated genes (UP) and down-regulated genes (DOWN) are listed separately.

Table S2. Gene ontology analysis of deregulated genes in *Hdac1*$^{\Delta/+n}$*Hdac2*$^{\Delta/\Delta n}$ (HD1s) and *Hdac1*$^{\Delta/\Delta n}$*Hdac2*$^{\Delta/\Delta n}$ (DKO) brains using DAVID software (http://david.abcc.ncifcrf.gov/).

Table S3. Primer sequences for genotyping and qRT-PCR.

Download Table S1

Download Table S2

Download Table S3
